# Depressive symptoms and functional dependence in near-centenarians and centenarians: a scoping review

**DOI:** 10.1186/s12877-026-07026-4

**Published:** 2026-02-06

**Authors:** Carla Gomes da Rocha, Armin von Gunten, Joëlle Rosselet Amoussou, Sofia Fernandes, Kim Uittenhove, Daniela S. Jopp, Olga Ribeiro, Henk Verloo

**Affiliations:** 1 School of Health Sciences, HES-SO Valais-Wallis, Sion, Switzerland; 2 Institute of Biomedical Sciences Abel Salazar, University of Porto, Porto, Portugal; 3 Service of Old Age Psychiatry, Site de Cery, Lausanne University Hospital, University of Lausanne, Prilly-Lausanne, Switzerland; 4 Medical Library-Cery, Site de Cery, Lausanne University Hospital and University of Lausanne, Prilly-Lausanne, Switzerland; 5 Maison de la Providence Nursing Home, Le Châble, Switzerland; 6 Institute of Psychology, University of Lausanne, Lausanne, Switzerland; 7 Swiss National Centre of Competence in Research LIVES, University of Lausanne, Lausanne, Switzerland; 8 RISE-Health, Nursing School, University of Porto, Porto, Portugal

**Keywords:** Centenarians, Near-centenarians, Depressive symptoms, Functional dependence, Activities of daily living, Scoping review

## Abstract

**Background:**

The growing population of centenarians faces unique health challenges. Of particular interest may be the co-occurrence of depressive symptoms and functional dependence, requiring comprehensive exploration.

**Objective:**

To map and summarize existing literature on depressive symptoms and functional dependence in near-centenarians and centenarians, focusing on prevalence rates, screening instruments, and the relationship between these two conditions.

**Inclusion criteria:**

Studies that explored depressive symptoms and functional dependence among individuals aged ≥ 95 years.

**Methods:**

This review was performed in accordance with the JBI Manual for Scoping Reviews. The PRISMA Extension for Scoping Reviews (PRISMA-ScR) standards were followed for reporting. The literature search was conducted in August 2023 in the following bibliographic databases: Embase.com, Medline ALL Ovid, CINAHL with Full Text, APA PsycInfo Ovid, Web of Science Core Collection, Cochrane Database of Systematic Reviews and Cochrane Central Register of Controlled Trials; including a grey literature search and citation tracking strategies.

**Results:**

Fifty-three studies from 1994 to 2023 were included, with 28.3% conducted in the USA. Most studies were quantitative and cross-sectional. Depressive symptom prevalence ranged from 10.5% to 73% among studies reported individually; the GDS–15 was the most commonly used instrument. Total functional dependence ranged from 20.8% to 30.3% (ADLs) and 27.5% to 63% (IADLs); the OARS Multidimensional Functional Assessment Questionnaire and the Katz Index were the most frequently employed instruments. Only 30.2% of studies explored the association between depressive symptoms and functional dependence, with 56.3% finding a significant relationship.

**Conclusions:**

The findings revealed geographical research disparities and underscored the need for diverse research methodologies for deeper insights into the health trajectories of the very old. Additionally, centenarians may not inherently have higher depressive symptoms than ‘younger’ seniors, suggesting possible resilience mechanisms. The relationship between depressive symptoms and functional dependence highlighted their mutual influence and potential to increase the risk of adverse outcomes.

**Implications:**

Diversifying research methodologies and expanding geographical scope are essential for a holistic understanding and international comparisons. Standardized guidelines for assessment instruments could facilitate consistent conclusions. Development and implementation of multifaceted interventions, such as preventive measures, professional competency enhancement, and caregiver support are central to addressing the needs of this population effectively.

**Supplementary Information:**

The online version contains supplementary material available at 10.1186/s12877-026-07026-4.

## Background

The 21st century is witnessing a dramatic increase in the population of centenarians. Predictions from the United Nations suggest an increase from half a million centenarians in 2015 to 3.4 million by 2050 and an astonishing 25 million by 2100 [1–3]. Europe is not an exception to this trend, with a notable increase in centenarians in recent decades. Census data from 2011 estimated that there were approximately 89,156 individuals aged 100 and over in 32 European countries [4], and this number has likely increased since then.

While the growth in the centenarian population underscores the positive strides made in medical advancements and improved quality of life, it might also give rise to concerns for both physical and mental well-being, potentially leading to unique health challenges. These may include the manifestation of depressive symptoms, maybe as a consequence but also a reason for functional dependence. In the absence of notable systematic investigation in very old age, the present paper aims at reviewing the actual findings to build a basis for further comprehensive investigation.

Aging trajectories can differ substantially between individuals. Some remain robust in physical, cognitive, and mental health domains, while others experience varying degrees of physical and cognitive decline [5, 6]. This variability brings forward significant challenges in understanding and addressing the specific needs of the very old, especially with an observed increase in multiple health conditions, including mental illnesses [7], within this growing population. Depression, in particular, stands out as a major public health concern in younger older age groups [7, 8], yet the situation is less clear in very old age.

As defined by the ICD-11, depression is characterized by a period of depressed mood or diminished interest in activities, occurring most of the day, nearly every day, for at least two weeks. This condition encompasses a range of symptoms including difficulty concentrating, feelings of worthlessness or guilt, hopelessness, changes in appetite or sleep, reduced energy or fatigue, and recurrent thoughts of death or suicide [9]. Crucially important, depression is not a normal part of aging, even at very advanced ages [10–12]. Clinically relevant depressive symptomatology has important negative consequences: it is associated with several risks, such as functional decline, falls and bone fractures, malnutrition, deterioration of other chronic conditions, delirium, increased risk of neurocognitive disorders, increased burdens on informal caregivers and healthcare professionals, frailty, institutionalization, and early death [13–17].

In the context of this review, the term ‘depressive symptoms’ may be more apt as it encompasses a broader spectrum of emotional states that can range from mild to severe. For older adults, manifestations of these symptoms might be subtle or concealed, making detection a challenge [18–21]. Yet, depressive symptoms in this age group often blur with what is perceived as ‘normal aging’, leading to frequent under-diagnoses [7, 19, 22–24]. Approximately half of these cases go unnoticed by healthcare professionals [14, 22] and when identified, about half remain untreated [14]. This discrepancy highlights the urgent need for further investigation and insight in this domain.

A factor that may be of central importance in the context of the developing depressive symptoms in very older age is the reduction of everyday life functional capacity, ultimately leading to becoming dependent. Functional dependence is defined as a reduction in a person’s capacity to perform basic activities of daily living (ADLs) or instrumental activities of daily living (IADLs) independently [25]. This condition is closely related to depressive symptoms and can emerge both as a potential risk factor and a consequence of these symptoms [10]. Understanding this complex relationship is of high importance. Particularly among the very old, several factors can influence the transition towards a state of functional dependence, making it difficult to determine whether they are the causes or consequences of depressive symptoms [25]. Studies have shown that functional dependence can intensify depressive symptoms [26, 27]. Depressive symptoms were also found to escalate the risk of functional dependence, impairing the ability to perform ADLs or IADLs [10, 26, 28]. Nevertheless, despite the importance of better understanding the development of both, functional impairment and depressive symptoms for quality of life, this reciprocal detrimental relationship does not seem to be among the priority domains of research on centenarians [13].

When trying to synthesize research investigating depressive symptoms and functional dependence, several methodological aspects need consideration, as they may have influenced the reported findings, including the age range covered in the studies, recruitment approach, or measures used. Therefore, exploring the characteristics of existing studies, including their origin, design, and sample demographics is crucial. Such details also provide insight into the broader context and help us understand cultural or demographic variations.

The present review aims at providing a comprehensive overview of studies on depressive symptoms and functional dependence in very old individuals. To the best of our knowledge, and based on a preliminary search across platforms including Embase.com, the Cochrane Database of Systematic Reviews, Epistemonikos and Prospero, no previous review has synthesized the available evidence on the prevalence of depressive symptoms and functional dependence among near-centenarians and centenarians. This work is part of a larger research initiative that seeks to identify the unique characteristics, challenges, and needs of centenarians [29].

Specifically, this review aims to map and summarize the available studies on these two conditions in near-centenarians and centenarians. It focused on the following questions:

1. What types of studies, based on their origin, research design, and key sample characteristics, have explored depressive symptoms and functional dependence in near-centenarians and centenarians?
2. What are the prevalence rates of depressive symptoms among near-centenarians and centenarians, and which instruments have been employed to screen for these symptoms?
3. What are the prevalence rates of functional dependence among near-centenarians and centenarians, and which instruments have been employed for its assessment?
4. Have any studies documented associations between depressive symptoms and functional dependence in this specific age group?

## Methods

This scoping review was carried out in accordance with the JBI Manual for scoping reviews [30]. The PRISMA Extension for Scoping Reviews (PRISMA-ScR) standards were followed for reporting [31].

We chose not to publish a protocol for this scoping review. Due to resource constraints, we prioritized immediate engagement with the review process over protocol publication.

### Selection criteria

To ensure a comprehensive understanding of the topics of interest, we established specific criteria to guide the inclusion of studies. Selection criteria regarding types of participants, the concepts of interest, the context of the studies, and the type of studies were as follows:

#### Types of participants

We considered studies that included near-centenarians and centenarians (i.e., individuals aged 95 or older).

#### Concepts of interest

This review primarily focuses on two conditions: depressive symptoms and functional dependence, as described below.

##### Depressive symptoms

For the purposes of this review, the term ‘depressive symptoms’ is used to capture the spectrum of signs and symptoms associated with depression [9]. The literature on the topic is vast, and various terms such as ‘low mood’, ‘depressive affect’, and ‘dysthymia’ are often used. Though these terms carry distinct nuances, for this review, any variations pointing towards a possible state of depression have been brought under the umbrella of ‘depressive symptoms’ and were considered eligible for inclusion (Additional file 1).

##### Functional dependence

‘Functional dependence’ is defined as an individual’s reduced capability to perform ADLs and/or IADLs without assistance [25]. Examples include bathing, dressing, toileting, using the telephone, preparing food, and housekeeping [32–34]. The literature frequently encompasses terms such as functional decline, disability, limitation, and incapacity. These are recognized as part of the broader dimension of functional health. Thus, for the scope of this review, all these variants were considered eligible for inclusion (Additional file 1).

#### Study contexts

Studies involving any subgroup of near-centenarians and centenarians were considered, irrespective of their living situation – community living, hospitalization, transitional care unit, or long-term care facilities (e.g., a nursing home). There were no geographical restrictions, meaning studies from any region or country were eligible.

#### Types of studies

Quantitative (experimental, quasi-experimental, and observational), qualitative, and mixed-methods studies were considered for inclusion in this scoping review. Comprehensive studies such as systematic reviews, meta-analyses, and meta-syntheses were also considered. Due to the potential limited depth of information, abstracts, commentaries, letters to the editor, and book reviews were excluded.

### Search strategy

The literature search was conducted in August 2023, in collaboration with a medical librarian (JRA), in six bibliographic databases: Embase.com, Medline ALL Ovid, CINAHL with Full Text, APA PsycInfo Ovid, Web of Science Core Collection, Cochrane Database of Systematic Reviews and Cochrane Central Register of Controlled Trials. All searches were conducted with no language or date restrictions. Additional searches were performed in Google Scholar, ProQuest Dissertations & Theses Global (PQDTGlobal), Dart Europe, and Open Grey, followed by backward and forward citation tracking of the included studies using the Citationchaser tool [35]. The detailed search strategies, keywords, and index terms are presented in Additional file 1.

### Screening and selection

Following the search, all identified records were uploaded into Endnote^®^ 20 [36]. Two reviewers (CGR and SF) independently checked the titles and abstracts for eligibility using Rayyan^®^ software [37]. Any discrepancies between them were resolved via discussions with a third reviewer (HV). The full texts of any relevant identified records were retrieved and analyzed to determine whether they met the inclusion criteria. Again, any issues were discussed and resolved via discussions with the third reviewer. The reasons for why specific full text records were excluded after analysis are provided in Additional file 2.

### Data extraction and reporting results

Data extraction was conducted by a single reviewer (CGR) using a standardized data extraction tool aligned with the review’s objectives and questions (Additional file 3). A 10% random sample of the included full-text records was independently checked by a second reviewer (HV) to ensure the reliability of the process. Any uncertainties or ambiguities were resolved in consultation with the research team. The data extracted encompassed details such as the year of publication, origin, research design, study objective(s), sample characteristics (sample size, mean age, living situation), concepts identified in relation to the conditions of interest, instruments used to screen for them, and the key findings relevant to the review questions (Additional file 3).

## Results

### Search results

**Fig. 1:**
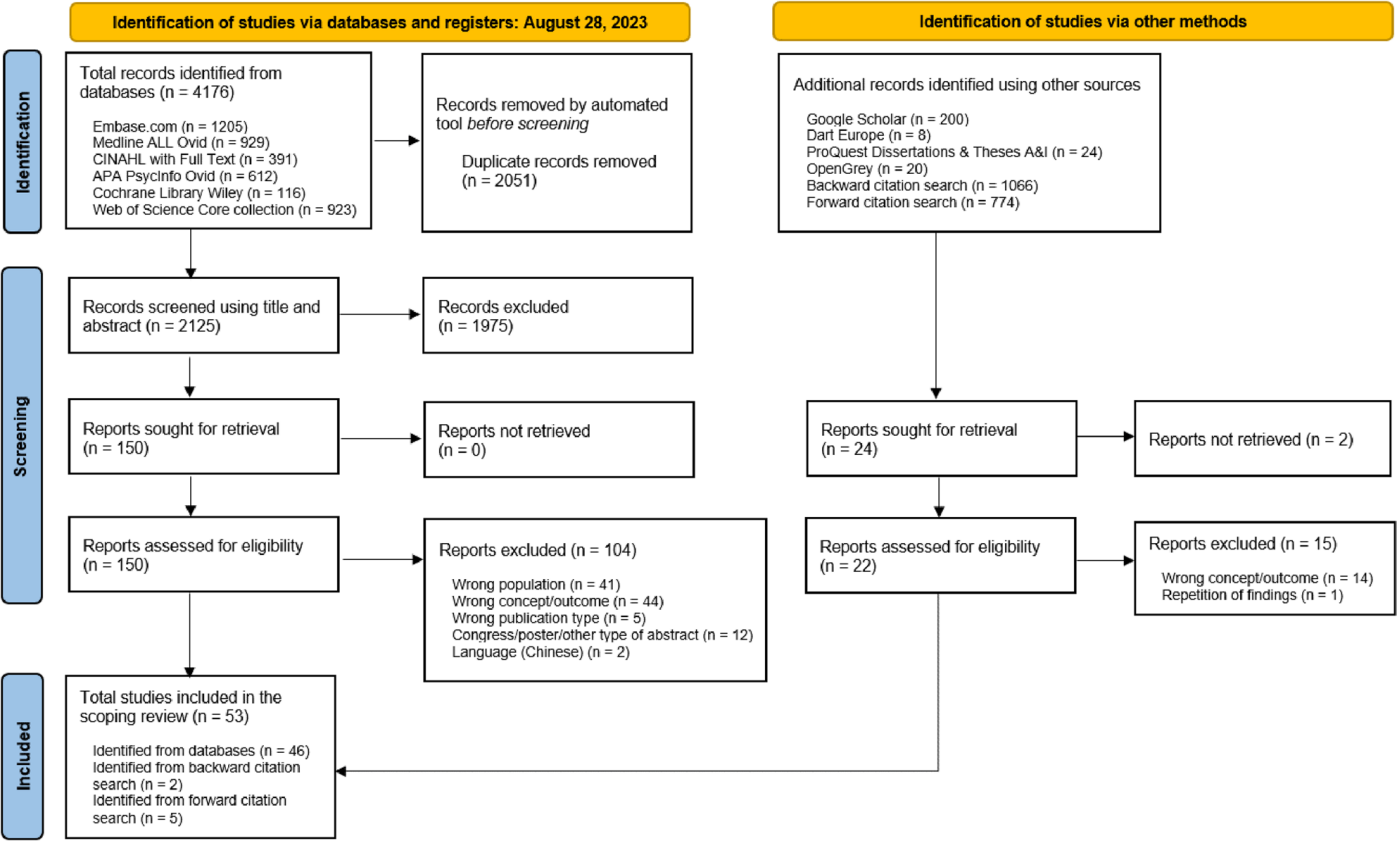
PRISMA 2020 Flow Chart: Search results and the study selection and inclusion process, following the presentation proposal by Page and colleagues [38].

Of 2125 records identified in the search and considered for eligibility, 150 references met our inclusion criteria based on title and abstract screening, and their respective full-text articles were then retrieved. Following full-text assessment, 53 articles met the inclusion criteria and were included in the scoping review (Fig. 1).

### Description of the studies retained

Our first research question focused on understanding the types of studies that have been published on depressive symptomatology and functional dependence among near-centenarians and centenarians. Important study characteristics were year of publication, geographic distribution (place of data collection), research designs employed (e.g., quantitative cross-sectional or longitudinal designs) and sample size and characteristics of the population (i.e., mean age, living situation) (Table 1).

**Table 1:** Characteristics of the studies retained (*n* = 53)

1st Author and year of publication	Country of origin	Study design	Objective(s)	Sample size	Age Metrics*	Concepts**	Living situation (context)***
Ailshire et al., 2011 [67]	U.S.A.	Quantitative, longitudinal	- Examine social characteristics and physical, functional, mental, and cognitive health.	*n* = 225 (follow-up)	- 96 y/o: *n* = 106 - 97 y/o: *n* = 102 - 98–100 y/o: *n* = 17	- Mental health - Functional limitations	- Community-based living: 64.8% - Institutionalized living: 37.4%
Araújo et al., 2016 [39]	Portugal	Quantitative, cross-sectional	- Identify subgroups in successful aging profiles; - Determine the roles of sociodemographic factors and psychological, social, and economic resources on successful aging.	*n* = 80	M = 101.01 ± 1.31 y/o	- Depression - Functional disability	- Community-based living: 68.8% - Institutionalized living: 31.3%
Bauco et al., 1996 [76]	Italy	Quantitative, cross-sectional	- Analyze physical and psychosocial variables based on a multidimensional conceptualization of health.	*n* = 109	M = 101.7 ± 1.96 y/o	- Depression level - Functional capacity (ADL independence)	Not reported
Boerner et al., 2019 [40]	Germany and Portugal	Quantitative, cross-sectional	- Examine how common thinking of and planning for end of life (EOL); - Examine whether patterns of EOL views are shaped by cultural and individual characteristics.	*n* = 218 • Germany: *n* = 87 • Portugal: *n* = 131	• Germany: M = 100.45 ± 0.52 y/o • Portugal: M = 101.21 ± 1.65 y/o	- Depressive symptoms - Functional ability	- Community-based living: • Germany: 34.5% • Portugal: 7% - Institutionalized living: • Germany: 34.5% • Portugal: 39.1%
Boerner et al., 2018 [41]	Germany	Quantitative, cross-sectional	- Explore how centenarians think about and plan for end of life (EOL); - Explore to what extent their proxy informants are aware of these thoughts.	*n* = 78	M = 100.46 ± 0.54 y/o Range: 99.16–103.34 y/o	- Depressive symptoms/depression - Functional ability	- Community-based living: 32.1% - Institutionalized living: 35.9%
Chen et al., 2020 [42]	China	Quantitative, cross-sectional	- Evaluate health status; - Investigate modifiable factors associated with health-related quality of life.	*n* = 876 (female subsample):	M = 95.26 ± 9.34 y/o	- Depression - Mobility and self-care	- Community-based living: 16.32%
Cheung & Lau, 2016 [43]	Hong Kong	Quantitative, cross-sectional	- Examine successful aging (SA); - Investigate whether SA is associated with biomedical and psychosocial-demographic factors.	*n* = 120	M = 97.66 ± 2.26 y/o Range: 95–108 y/o	- Depressive symptoms - Functional independence	- Community-based living: 85% - Institutionalized living: 15%
Cimarolli & Jopp, 2014 [68]	U.S.A.	Quantitative, cross-sectional	- Document the prevalence of self-reported vision, hearing, and dual sensory impairment; - Explore associations between these impairments and functional disability.	*n* = 119	M = 99 y/o Range: 95–107 y/o	- Depressive symptomatology - Functional disability	- Community-based living: 49.6% - Institutionalized living: 19.3%
Cimarolli et al., 2018 [44]	U.S.A.	Quantitative, cross-sectional	- Examine the separate and combined effects of self-reported vision and hearing impairment on depressive symptoms.	*n* = 119	M = 99 y/o Range: 95–107 y/o	- Depressive symptomatology - Functional disability	- Community-based living: 73.9%
Clayton et al., 1994 [69]	U.S.A.	Quantitative, cross-sectional	- Examine differences between rural and urban participants across the following variables: physical health, ADLs, mental health, and life satisfaction.	*n* = 84 • Rural: *n* = 18 • Urban: *n* = 66	Range (urban participants): 100–106 y/o	- Mental health - Functional Health	Community-based living sample (only community-dwelling individuals were recruited)
Cohen-Mansfield et al., 2013 [88]	Israel	Quantitative, cross-sectional (+ longitudinal)	- Examine whether old age, old-old age, and oldest-old age comprise distinct categories of demographics, health, function, and well-being.	*n* = 77	M = 97.8 ± 3 y/o Range: 95–108 y/o	- Depressed affect (as one of the indicators of “well-being”) - ADLs and IADLs (as indicators of “function”)	- Institutionalized living: 24.7%
Davey et al., 2010 [70]	U.S.A.	Quantitative, cross-sectional	- Provide normative data on cognitive functioning and physical performance, health and health behaviors, and diseases.	*n* = 244	M = 100.5 y/o Range: 98–108 y/o	- Depression - Physical functional capacity	- Community-based living: 37.3% - Institutionalized living: 62.7%
Deckers et al., 2018 [45]	U.K.	Quantitative, longitudinal	- Investigate associations between modifiable risk and protective factors and severe cognitive impairment and dementia.	*n* = 119 • Group 1 “*Incident severe cognitive impairment status*”: *n* = 44 • Group 2“*Incident dementia/severe cognitive impairment status*”: *n* = 75	- Group 1 (at death): M = 96.7 ± 3.9 y/o - Group 2 (at death): M = 95.1 ± 3.9 y/o	- Depression - Disability in ADLs and IADLs	- Group 1: • Community-based living: 93.2% • Institutionalized living: 6.8% - Group 2: • Community-based living: 92% • Institutionalized living: 8%
Dello Buono et al., 1998 [77]	Italy	Quantitative, cross-sectional	- Assess the quality of life.	*n* = 38	M = 101.13 ± 1.52 y/o	- Symptoms of depression - ADLs and IADLs	- Community-based living: 63.2% - Institutionalized living:36.8%
Feng et al., 2023 [46]	China	Quantitative, cross-sectional (+ longitudinal)	- Investigate the effects of physical inability, depression and cognitive impairment on the prognosis of centenarians.	*n* = 170	MED = 102 y/o IQR = 101–104 y/o	- Depression - Physical inability	Not reported
Freeman et al., 2017 [47]	Canada	Quantitative, cross-sectional	- Describe patterns of health deficits in participants receiving formal care.	*n* = 3391	M = 101.5 ± 1.9 y/o Range: 100–114 y/o	- Depressive symptoms - Impaired physical functioning	- Community-based living: 34.3% - Institutionalized living: 65.7%
Galenkamp et al., 2013 [78]	The Netherlands	Quantitative, longitudinal	- Investigate changes in self-rated health; - Examine its sensitivity to changes in chronic conditions and functioning.	*n* = 21 (wave 4)	MED = 99 y/o Range: 99–106 y/o	- Depression - Functional status	- Community-based living: 61.9%
Garasky et al., 2012 [71]	U.S.A.	Quantitative, cross-sectional	- Explore whether economic status is related to: (a) ability to manage ADLs; (b) mental abilities/cognition; (c) psychological well-being/depression; (d) ability to handle finances; - Explore whether these relationships vary by institutionalization status.	*n* = 152	M = 101.6 y/o	- Depressive symptoms - ADLs/functional ability	- Institutionalized living: 81.5%
Herr et al., 2018 [48]	Japan, France, Switzerland, Denmark, and Sweden	Quantitative, cross-sectional	- Estimate the prevalence of frailty and analyze associated factors.	*n* = 1228	- Minimum age: 100 y/o	- Depression - Disability for ≥ 2 ADL	- Institutionalized living: 47.6%
Infusino et al., 1996 [79]	Italy	Quantitative, cross-sectional	- Evaluate the clinical and socio-economic conditions (multidimensional investigation).	*n* = 40	M = 102.3 ± 1.8 y/o Range: 100–107 y/o	- Mood/depression - Functional status/activity	- Community-based living: 90%
Jang et al., 2004 [72]	U.S.A.	Quantitative, cross-sectional	- Explore factors responsible for individual differences in experiences with disease and disability.	*n* = 72	M = 100.7 ± 1.29 y/o	- Depressive symptoms - Disability	Community-based living sample (only community-dwelling individuals were recruited)
Jopp et al., 2016 [49] ^a^	U.S.A.	Quantitative, cross-sectional ^a^	- Examine the challenges experienced by very old individuals and their consequences for well-being and mental health.	*n* = 75	M = 99.01 ± 2.59 y/o Range: 95–107 y/o	- Depressive symptoms - ADL restrictions/disability	- Community-based living: 71% - Institutionalized living: 29%
Jopp, Park et al., 2016 [12]	U.S.A.	Quantitative, cross-sectional	- Provide a comprehensive picture of key domains of functioning (physical, cognitive, social, and mental function); - Determine predictors of mental health indicators.	*n* = 119	M = 99.25 y/o Range: 95–107 y/o	- Depressive symptoms - Functional health	- Community-based living: 74% - Institutionalized living: 26%
Lau & Cheung, 2016 [50]	Hong Kong	Quantitative, cross-sectional	- Understand successful aging especially at the extreme of longevity.	*n* = 153	M = 97.7 ± 2.4 y/o Range: 95–108 y/o	- Depression - Functional capacity	- Community-based living: 81% - Institutionalized living: 19%
Li et al., 2022 [51]	China	Quantitative, cross-sectional	- Investigate the relationships between dietary diversity scores and anxiety and depression.	*n* = 38	MED = 101 y/o IQR = 100–102 y/o	- Depression - Dependence	Not reported
Madrigal-Leer et al., 2020 [52]	Costa Rica	Quantitative, cross-sectional	- Describe clinical, functional, mental, and social profiles.	*n* = 43	M = 101.93 y/o Range: 100–107 y/o	- Depression - Functional performance	- Community-based living: 95.4% - Institutionalized living: 4.6%
Margrett et al., 2010 [73]	U.S.A.	Quantitative, cross-sectional	- Examine how cognitive ability and personality explain depression.	*n* = 158	M = 99.82 y/o Range: 98–109 y/o	- Depressive symptoms - Functional status	- Community-based living: 49.6% - Institutionalized living: 50.4%
Martin et al., 2012 [74]	U.S.A.	Quantitative, cross-sectional	- Understanding the determinants of exceptional longevity above and beyond health outcomes.	*n* = 152 (Phase 1)	M = 101.6 ± 2.13 y/o Range: 100 - 112 y/o	- Depression - Functional capacity/functional health	- Institutionalized living: 81.5%
Martin et al., 2000 [75]	U.S.A.	Quantitative, cross-sectional	- Assess levels of depression and predictors of depressive symptoms.	*n* = 136	M = 100.7 y/o Range: 99–110 y/o	- Depression - ADLs	Community-based living sample (only community-dwelling individuals were recruited)
Molander et al., 2010 [80]	Sweden and Finland	Quantitative, cross-sectional	- Investigate the relationship between blood pressure and cognitive impairment.	*n* = 158	Not specified	- Depressive symptoms/depression - Dependence in ADLs	- Community-based living: 35.3% - Institutionalized living: 64.7%
Niimura et al., 2020 [53]	Japan	Quantitative, longitudinal	- Examine the sociopsychological characteristics of the oldest-old in Japan.	*n* = 26 (group “*Full questionnaire with in-home interview and exam*”)	M = 97.7 ± 2.1 y/o	- Depression - Functional capacity	- Community-based living: 88.5% - Institutionalized living: 11.5%
Nyqvist et al., 2006 [81]	Sweden	Quantitative, cross-sectional	- Measure social capital in the oldest old, and its association with different dimensions of health.	Not specifically reported for the 95 + group	Not reported	- Depressive symptoms - Functional ability	Not specifically reported for the 95 + group
Oseland et al., 2016 [54]	U.S.A.	Quantitative, cross-sectional	- Test how traumatic life events affect the outcomes of physical disability, perceived health impairments, depressive symptoms, and social support.	*n* = 154	M = 101 ± 1.71 y/o	- Depressive symptoms - Functional health	Community-based living sample (only community-dwelling individuals were recruited)
Pedro et al., 2017 [55]	Mexico	Quantitative, cross-sectional	- Describe demographic characteristics and health status.	*n* = 393	M = 101.82 ± 2.02 y/o Range: 100–112 y/o	- Depression - Functional dependence	- Community-based living: 95.4% - Institutionalized living: 4.6%
Rabunal Rey et al., 2004 [82]	Spain	Quantitative, cross-sectional	- Establish the social, functional and medical status of centenarians.	*n* = 54	M = 100.9 ± 1.3 y/o	- Depression - Functional status	- Community-based living: 87%
Ravaglia et al., 1997 [83]	Italy	Quantitative, cross-sectional	- Evaluate physical ability and psychocognitive status regarding sociodemographic, behavioral, and biomedical variables.	*n* = 84	M = 97.4 ± 3.1 y/o Range: 90–106 y/o	- Symptoms of depression - Physical ability	- Community-based living: 37% - Institutionalized living: 63%
Ribeiro et al., 2018 [56]	Portugal	Quantitative, cross-sectional	- Characterize the frailty phenotype and the relationships between frailty and depressive symptoms; - Explore the most characteristic depressive symptoms of the frailty syndrome.	*n* = 91	M = 101.0 ± 1.3 y/o Range: 100–105 y/o	- Depression - Functionality/functional status	- Community-based living: 60.5% - Institutionalized living: 39.5%
Richmond et al., 2011 [90]	Australia	Quantitative, cross-sectional	- Examine quality of life, social, cognitive, and physical function, anxiety and depression, and their associations.	*n* = 188	− 100 y/o: *n* = 93 − 101 y/o: *n* = 39 − 102 y/o: *n* = 18 − 103 y/o: *n* = 18 − 104 y/o: *n* = 7 - ≥ 105 y/o: *n* = 12	- Depression - Physical functioning	- Community-based living: 48% - Institutionalized living: 52%
Schroyen et al., 2020 [57]	France	Quantitative, cross-sectional	- Hypothesis: people with sensory impairments (hearing and vision) and disabilities would feel older (subjective age).	*n* = 75	M = 95.8 ± 2.6 y/o	- Depressive symptomatology/depression - Disability	Community-based living sample (only community-dwelling individuals were recruited)
Song et al., 2022 [58]	China	Quantitative, cross-sectional	- Explore the relationship between nutritional status and depression.	*n* = 1002	MED = 102 y/o IQR = 101–104 y/o	- Depression - Functional status of daily activities	Not reported
Struckmeyer et al., 2021 [59]	U.S.A.	Quantitative, cross-sectional	- Examine the influences of early and recent traumatic life experiences on loneliness.	*n* = 154	M = 101.01 ± 1.72 y/o Range: 99–109 y/o	- Depressive affect - Functional health	- Community-based living: 44.8% - Institutionalized living: 55%
Sun et al., 2023 [60]	China	Quantitative, cross-sectional	- Explore the possible associations between cardiac structure and function and depressive disorder.	*n* = 682	M = 102.4 ± 2.7 y/o Range: 100–116 y/o	- Depressive disorder - Physical disability	Not reported
Tafaro et al., 2005 [84]	Italy	Quantitative, cross-sectional	- Determine which are the survival determinants using a neural network.	*n* = 110	M = 101.6 ± 1.8 y/o Range: 100–108 y/o	- Depression/mood - Functional status/self-sufficiency	- Community-based living: 7.5%
Tafaro et al., 2002 [85]	Italy	Quantitative, longitudinal	- Describe the relationship between aging and depression.	*n* = 157	M = 101.7 ± 1.8 y/o Range: 100–108 y/o	- Depression status - Daily activities	Unclear
Teixeira et al., 2019 [61]	Portugal	Quantitative, cross-sectional	- Explore the recent history of falls, the existence of a fear of falling, and their associated factors.	*n* = 109	M = 101.0 ± 1.5 y/o Range: 100–107 y/o	- Depression - Functional disability	- Community-based living: 66.1% - Institutionalized living: 33.9%
Tindale et al., 2019 [62]	Canada	Quantitative, longitudinal	- Determine whether super-seniors showed compression of morbidity; - Test whether the allele frequencies of longevity-associated variants (APOE and FOXO3) were more extreme in this population.	*n* = 13 (second interview)	M = 100.1 ± 3.3 y/o Range: 96–106 y/o	- GDS score - IADL score	Unclear
Urciuoli et al., 1998 [86]	Italy	Quantitative, cross-sectional	- Compare quality of life among participants living in nursing homes and at home.	*n* = 66	M = 95.5 ± 6.8 y/o	- Depression - Functioning	- Community-based living: 56.1% - Institutionalized living: 43.9%
von Heideken Wågert et al., 2006 [87]	Sweden	Quantitative, cross-sectional	- Describe health status and living conditions; - Estimate age and sex differences.	*n* = 72	Range: 95–103 y/o	- Depression - Independence in ADLs	- Community-based living: 21% - Institutionalized living: 79%
Wang et al., 2020 [63]	China	Quantitative, cross-sectional	- Investigate the sociodemographic characteristics, health profiles, and social relationships.	*n* = 100	− 100–105 y/o: *n* = 80 - > 105 y/o: *n* = 20	- Feelings of depression - Independence	Community-based living sample (only community-dwelling individuals were recruited)
Yang, 2013 [89] ^b^	Taiwan	Quantitative, cross-sectional ^b^	- Explore participants’ living conditions and their care and support systems.	*n* = 100	M = 102.54 y/o Range: 100–110 y/o	- Depression/depressive symptoms - Functioning	- Community-based living: 72% - Hospital: 1% - Institutionalized living: 27%
Yao et al., 2018 [64]	China	Quantitative, cross-sectional	- Evaluate the relationship between vitamin D levels and depressive symptoms.	*n* = 940	M = 102.5 ± 2.7 y/o Range: 100–115 y/o	- Depressive symptoms/depression - ADLs	- Community-based living: 85.7%
Zaccaria et al., 2022 [65]	U.S.A.	Quantitative, cross-sectional	- Investigate the prevalence of combinations of social isolation and loneliness.	*n* = 94	M = 99.6 ± 2.4 y/o Range: 95–107 y/o	- Depressive symptomatology - Functional health	- Community-based living: 78.7% - Institutionalized living: 21.3%
Zhang et al., 2022 [66] ^b^	China	Psychometric validation study ^b^ (cross-sectional analysis)	- Evaluate the psychometric properties of the GDS-15 scale and a simplified version of it.	*n* = 838	M = 102.48 ± 2.74 y/o	- Depressive symptoms - Physical function	- Community-based living: 99.0% - Institutionalized living: 1%

* Age metrics: Mean ± SD; median with IQR; distribution (counts) by age category; minimum age

** Concepts of interest: as defined in the respective studies

*** Living situation (context): Community-based living: refers to settings such as private homes, apartments, retirement villages, etc. Institutionalized living: refers to places like nursing homes, long-term care facilities, and group homes for dementia patients

^a^This study employed theme-based coding for response categorization and quantification

^b^These studies adopted a multi-methods approach (quantitative and qualitative techniques)

#### Year of publication

The 53 selected studies were published over a period ranging from 1994 to 2023. It is worth noting that a surge in research occurred between 2016 and 2023, as 54.7% (*n* = 29) of the studies were published during this time frame [12, 39–66]. For a visual representation of the distribution of the retained studies by 5-year periods, refer to Additional file 4.

#### Geographic distribution

Research on the topic spans four continents: North America (*n* = 19, 35.8%), including two studies conducted in Latin America [12, 44, 47, 49, 52, 54, 55, 59, 62, 65, 67–75], Europe (*n* = 19, 35.8%) [39–41, 45, 56, 57, 61, 76–87], Asia (*n* = 13, 24.5%) [42, 43, 46, 50, 51, 53, 58, 60, 63, 64, 66, 88, 89], and Australia (*n* = 1, 1.9%) [90], whereas no studies were conducted in Africa or South America. Specific countries, such as the USA, China, and Italy, are more frequently represented. The USA accounts for 15 (28.3%) studies [12, 44, 49, 54, 59, 65, 67–75], China for eight (15.1%) [42, 46, 51, 58, 60, 63, 64, 66], and Italy for seven (13.2%) [76, 77, 79, 83–86]. Additional research has been conducted in various other countries, providing a diverse geographic perspective on the issue.

For a visual representation of the geographic distribution of the studies included in this review, refer to Additional file 5.

#### Study design

Methodologically, all 53 studies are rooted in a quantitative approach. Yet, within this group, three (5.7%) studies also integrated qualitative techniques, such as theme-based coding to categorize and quantify responses [49, 66, 89]. In terms of design, 47 (88.7%) of the studies adopted a cross-sectional design or reported results of cross-sectional analysis within a broader longitudinal study, whereas six (11.3%) studies employed a longitudinal design [45, 53, 62, 67, 78, 85] (Table 1).

#### Sample size and age characteristics

The sample sizes in the retained studies showcase a wide variability, ranging from as few as 13 participants [62] to as many as 3391 [47] (Table 1). The pooled mean age across these studies is 100.7 years, underscoring the specific focus on the near-centenarian and centenarian populations.

#### Living situation of the participants (context)

Of the studies reviewed, 28 (52.8%) recruited participants from two main settings: private residences (community-based living) and nursing homes (institutionalized living) [12, 39–41, 43, 45, 47, 49, 50, 52, 53, 55, 56, 59, 61, 65–68, 70, 73, 77, 80, 83, 86, 87, 89, 90]. A subset of six (11.3%) studies specifically focused on participants solely from the community-based living category [54, 57, 63, 69, 72, 75]. It is of importance to highlight that none of the retained studies recruited participants from hospitals, with one exception, yet hospital residents only represented 1% of its participants [89] (Table 1).

### Depressive symptoms in near-centenarians and centenarians: prevalence rates and instruments used

To address our second research question, we explored the prevalence rates of depressive symptoms and the instruments employed to screen for them in near-centenarian and centenarian populations.

Depressive symptoms in the reviewed studies were examined in two primary ways: by prevalence rates and by mean scores. Regarding prevalence rates, our reporting approach differed based on how frequently a screening instrument was used. If an instrument was employed in only one study, we presented its results individually. For instruments used in multiple studies, we grouped the results. Prevalence rates for studies reported individually ranged from approximately 10.5% [45] to 73.0% [67]. The computed median prevalence, derived from grouped studies, fell within this range, ranging from approximately 12.7% [48, 70, 82] to 27.1% [12, 50, 51, 55, 58, 60, 64, 80, 87], as can be seen in Table 2. For a visual representation of the prevalence of depressive symptoms, refer to Additional file 6.

**Table 2:** Instruments employed to screen for depressive symptoms with corresponding prevalence rates and/or associated scores (*n* = 50)

Instruments		Number of studies using the instrument *n* (%)	Studies (1st author and year of publication)	Prevalence data^*^	Scores	
					Score data^**^ (points)	Scoring strategy/interpretation
GDS	GDS-15	21 (42.0%)	Cheung & Lau, 2016 [43]; Feng et al., 2023 [46]; Jopp, Park et al., 2016 [12]^**a**^; Lau & Cheung, 2016 [50]; Li et al., 2022 [51]; Molander et al., 2010 [80]; Pedro et al., 2017 [55]; Song et al., 2022 [58]; Sun et al., 2023 [60]; von Heideken Wågert et al., 2006 [87]; Yao et al., 2018 [64]	MED = 27.1% (Range: 13.2% − 38.0%)	**§**	**§**
			Araújo et al., 2016 [39]^**†**^; Cimarolli & Jopp, 2014 [68]; Cimarolli et al., 2018 [44]; Jopp, Park et al., 2016 [12]; Lau & Cheung, 2016 [50]; Margrett et al., 2010 [73]; Niimura et al., 2020 [53]^**b**^; Nyqvist et al., 2006 [81]; Pedro et al., 2017 [55]; Sun et al., 2023 [60]; Teixeira et al., 2019 [61]^**†**^; Tindale et al., 2019 [62]; von Heideken Wågert et al., 2006 [87]; Yao et al., 2018 [64]; Zaccaria et al., 2022 [65]; Zhang et al., 2022 [66]	**§**	MED of Means: 4.4 (Range: 2.2–5.3) Prop: 0.29 (29%)	• 15 items • 2-point scale (0 = no, 1 = yes, with some reversed items) • Possible score range (sum): 0 − 15 • Score interpretation: • 0–4: normal • 5–9: mild • 10–15: severe Note: Lau & Cheung (2016) (50) considered scores > 5 as indicative of a depressive tendency, while Sun et al. (2023) (60) considered scores > 6 as indicative of a depressive disorder.
	GDS-30	10 (20.0%)	Infusino et al., 1996 (79); Martin et al., 2012 [74]; Martin et al., 2000 [75]; Ravaglia et al., 1997 [83]; Tafaro et al., 2002 [85]; Yang, 2013 [89]	MED = 22.5% (Range: 0% − 45.7%)	**§**	**§**
			Bauco et al., 1996 [76]; Garasky et al., 2012 [71]; Jang et al., 2004 [72]; Tafaro et al., 2005 [84]; Tafaro et al., 2002 [85]	**§**	MED of Means: 10.1 (Range: 8.2–10.4) Prop: 0.34 (34%)	• 30 items • 2-point scale (0 = no, 1 = yes, with some reversed items) • Possible score range (sum): 0 − 30 • Score interpretation: • 0–9: normal • 10–19: mild • 20–30: severe
		1 (2.0%)	Clayton et al., 1994 [69]^**†**^	**§**	M = 37.4	• 30 items • 2-point scale (1 = yes, 2 = no, with some reversed items) • Possible score range (sum) and score interpretation: unclear
	GDS^**c**^	1 (2.0%)	Madrigal-Leer et al., 2020 [52]^**c**^	40.5%	**§**	**§**
	GDS-14	1 (2.0%)	Ribeiro et al., 2018 [56]	35.2%	M = 4.5 Prop = 0.32 (32%)	• 14 items • 2-point scale (0 = no, 1 = yes, with some reversed items) • Possible score range (sum): 0 − 14 • Score interpretation: Scores ≥ 6 indicate possible depression
	GDS-10	1 (2.0%)	Boerner et al., 2018 [41]	**§**	M = 2.1 Prop = 0.21 (21%)	• 10 items • 2-point scale (0 = no, 1 = yes) • Possible score range (sum): 0 − 10 • Score interpretation: Higher scores indicated higher depressive symptomatology
	GDS-5	1 (2.0%)	Boerner et al., 2019 [40]^**†**^	**§**	M = 0.3 Prop = 0.06 (6%)	• 5 items • 2-point scale (0 = no, 1 = yes) • Possible score range (sum): 0 − 5 • Score interpretation: Higher scores indicated higher depressive symptomatology
CES-D	CES-D	3 (6.0%)	Cohen-Mansfield et al., 2013 [88]; Niimura et al., 2020 [53]^**b**^	**§**	MED of Means^**f**^ = 5.6 (Range: 0.8–10.4)	• 20 items • 4-point scale (0 = none of the time/rarely, 3 = most/all of the time) • Possible score range (sum): 0–60 • Score interpretation: Higher scores indicate increased symptomatology
			Schroyen et al., 2020 [57]	14.7%	**§**	**§**
	CES-D – brief version	1 (2.0%)	Ailshire et al., 2011 [67]	73%	M = 1.8	• 8 items • 4-point scale (“0 = none of the time or rarely” to “3 = most or all of the time”) • Possible score range (sum): 0–24 • Score interpretation: Higher scores indicate increased depressive symptoms
BSI (Depression dimension)		2 (4.0%)	Dello Buono et al., 1998 [77]^**d**^; Urciuoli et al., 1998 [86]^**†**^	**§**	MED of Means^**f**^ = 0.6 (Range: 0.6–0.7)	• 6 items • 5-point rating scale (“0 = not at all” to “4 = extremely”) • Possible score range (sum): 0–24 • Score interpretation: Higher scores indicated greater disturbance by certain symptoms.
EQ-5D-3 L		1 (2.0%)	Chen et al., 2020 [42]	13.8%	**§**	**§**
CAMDEX		1 (2.0%)	Deckers et al., 2018 [45]^**†**^	10.5%	**§**	**§**
DRS		1 (2.0%)	Freeman et al., 2017 [47]^**†**^	19.1%	**§**	**§**
HADS (depression subscale)		1 (2.0%)	Richmond et al., 2011 [90]	13.5%	M = 4.1	• 7 items • 4-point scale (“0” to “3”, with answering options varying per item) • Possible score range (sum): 0–21 • Score interpretation: • 0–7: normal • 8–10: mild • 11–15: moderate • 16–21: severe
Doctor-diagnosed depression or medical history assessment		3 (6.0%)	Davey et al., 2010 [70]; Herr et al., 2018 [48]; Rabunal Rey et al., 2004 [82]	MED = 12.7% (Range: 11.1% − 14.0%)	**§**	**§**
One question: “*Has your physician told you that you have depression/depressiveness?”*		1 (2.0%)	Galenkamp et al., 2013 [78]	18.8%	**§**	**§**
One question: “*Did you feel upset or depressed in the past 30 days?*”		1 (2.0%)	Wang et al., 2020 [63]	11.0%	**§**	**§**

*BSI* Brief Symptom Inventory,* CAMDEX* Cambridge Mental Disorders in the Elderly Depressive Symptoms Scale,* CES-D* Center for Epidemiologic Studies Depression Scale, *DRS* Depression Rating Scale, from the interRAI-Home Care Assessment instrument, *EQ-5D-3L* Three-Level EuroQol-5D Scale,* GDS* Geriatric Depression Scale,* HADS* Hospital Anxiety and Depression Scale

*Prevalence data: 'MED' = median prevalence rate for multiple studies using the same instrument. 'Range' = range of prevalence rates for studies using the same instrument

**Score Data (Means/Proportion for GDS, if applicable): 'M' = mean score. 'MED of Means' = median of mean scores across studies using the same instrument. 'Prop' = proportion of positively responded items (calculated for different GDS versions due to its "yes" or "no" response format; expressed both as a decimal and a percentage)

^§^Data that were either not reported by the authors or are not applicable

**†** Studies describing depressive symptoms in different subgroups (e.g., home vs. nursing homes). Combined mean prevalence rates and/or scores were computed for this table

^a^Prevalence rate from Jopp, Park *et al*. (2016) [12] was an estimated 20% (“Over 80% of the sample did not meet the criteria for clinical depression”)

^b^The study by Niimura *et al*. (2020) [53]employed two screening instruments for depressive symptoms (GDS-15 and CES-D)

^c^Madrigal-Leer *et al*. (2020) [52] did not specify which GDS version was used (unknown GDS version)

^d^Clayton *et al*. (1994) [69]did not specify which GDS version was used. Based on their citations, we inferred the initial 100-item version was employed, which led to the GDS-30 afterwards

^e^Dello Buono et al. (1998) [77] also employed the Depression and Anxiety Scale from the LEIPAD Quality of Life Assessment Instrument. However, it was excluded from this review as it also pertains to anxiety

For the mean scores, we adopted a grouping approach analogous to our method for prevalence. In the case of the GDS, across all its versions, we not only presented the computed median of mean values but also showcased the proportion of positively responded items in Table 2. This approach facilitates a more comprehensive understanding and allows for straightforward comparisons across the various GDS versions. Specifically, among the GDS versions we mapped, the proportions of positively responded items ranged between approximately 21% (GDS-10) and 34% (GDS-30), with the exception of the GDS-5 which stood notably lower at approximately 6%.

A total of 10 instruments (or approaches) for screening depressive symptoms were identified across the included studies and are presented in Table 2, which lists the instruments reported in 50 of the 53 included studies (three studies were omitted from the Table as they did not specifically report prevalence data on depressive symptoms [49, 54, 59]; however, they were included in the review due to their exploration of associations between depressive symptoms and functional dependence). The GDS – 15 was the most utilized instrument, featuring in 21 (42.0%) of the included studies. It is important to note that the use of the GDS in these studies primarily underlines its role as an assessment tool. Indeed, none of the studies reported using criteria from the Diagnostic and Statistical Manual of Mental Disorders (DSM) or the International Classification of Diseases (ICD) for a more comprehensive diagnostic process. For a visual representation of the identified instruments to screen for depressive symptoms, refer to Additional file 7.

For the majority of the instruments, all scores aligned with the established cut-offs for either normal or mild symptoms of depression except for the version of the GDS-30 used by Clayton et al. [69] (Table 2).

Additionally, four (8.0%) of the 50 studies not only employed a screening tool for depressive symptoms but also reported having investigated participants’ past and current experiences with depression. The sources of this complementary information included health care professionals, medical charts, proxy relatives, or direct clinical assessments by psychiatrists or geriatricians [52, 60, 87, 90]. Moreover, three (6.0%) studies focused exclusively on depression diagnosis provided by a physician or medical history assessment [48, 70, 82]. One (2.0%) study inquired about participants’ history of physician-diagnosed depression (self-reported data) [78], and another study (2.0%) probed whether participants felt upset or depressed in the previous 30 days [63].

### Functional dependence in near-centenarians and centenarians: prevalence rates and instruments used

In response to our third research question, we explored the prevalence rates of functional dependence and the instruments used to assess this issue among near-centenarians and centenarians.

In the reviewed studies, functional dependence was analyzed in two primary ways, mirroring the approach taken with depressive symptoms: through prevalence rates and mean scores. When presenting prevalence data, our approach varied based on the frequency with which a particular screening instrument was employed. For instruments that were employed in a single study, we reported the results individually. For instruments used across multiple studies, we grouped their results.

Regarding ‘functional dependence’ in ADLs, rates from studies reported individually varied between approximately 58% and 82% [67]. In one study, restrictions in performing ADLs emerged as the most frequently cited challenge by participants, with a rate of 42.7% [49]. The computed median prevalence, obtained from grouped studies, ranged between approximately 50.6% [46, 51, 60, 64, 66, 82] and 57.4% [50, 55]. ‘Total functional dependence’ in ADLs—indicating complete reliance on others for all ADLs—was captured in one study at a rate of 29% [89]. The computed median prevalence derived from grouped studies for this category varied from approximately 20.8% [52, 82] to 30.3% [77, 79, 83, 90] (Table 3).

**Table 3:** Instruments employed to assess functional dependence with corresponding prevalence rates and/or associated scores (*n* = 48)

Instruments	Number of studies using the instrument *n* (%)	Studies (1st author and year of publication)	Prevalence data^*^	Scores	
				Score data^**^ (points)	Scoring strategy/interpretation
OARS	12 (25.0%)	Araújo et al., 2016 [39]^**†**^; Boerner et al., 2019 [40]*; Boerner et al., 2018 [41]; Clayton et al., 1994 [69]^**†**^; Davey et al., 2010 [70]; Jopp, Park et al., 2016 [12]; Teixeira et al., 2019 [61]	**§**	ADL: MED of means = 9.6 (Range: 8.6–12.6)	**ADL: 7 items; IADL: 7 items;** • Rating: 3-point scale (0 = can’t do without help to 2 = no difficulty) • Score range: 0–14 (ADL & IADL each) • Interpretation: Higher scores indicated greater independence
		Araújo et al., 2016 [39]^**†**^; Boerner et al., 2019 [40]^**†**^; Boerner et al., 2018 [41]; Clayton et al., 1994 [69]^**†**^; Davey et al., 2010 [70]; Jopp, Park et al., 2016 [12]; Teixeira et al., 2019 [61]; Zaccaria et al., 2022 [65]	**§**	IADL: MED of means = 7.2 (Range: 4.3–10.0)	
		Cimarolli & Jopp, 2014 [68]; Cimarolli et al., 2018 [44]	**§**	ADL + IADL: MED of means = 26.7 *(same value for both studies)*	**ADL + IADL: 14 items;** • Rating: 4-point scale (1 = no difficulty to 4 = can’t do without help) • Score range: 14–56 • Interpretation: Higher scores indicated greater dependence
		Jang et al., 2004 [72]	**§**	ADL + IADL: M = 7.1	**ADL + IADL: 14 items;** • Rating: 3-point scale (0 = can do without help to 2 = unable to do) • Score range: 0–28 • Interpretation: Higher scores indicated greater dependence.
		Jopp, Park et al., 2016 [12]	• 40.0% had difficulties with 1 or 2 ADLs. • 28.0% were independent in ADLs; 17.0% were independent in IADLs. • ADL challenges: taking a bath (55.0%), getting dressed (52.0%), moving in and out of bed (48.0%). • IADL challenges: light housework (77.0%), shopping (76.0%), preparing meals (65.0%), getting around/traveling (64.0%).	**§**	**§**
		Martin et al., 2012 [74]	• 50.0% were still able to leave their homes. • < 10.0% could go to various locations or shop without assistance. • Activities like cooking, housework, and managing money were very difficult.	**§**	**§**
Katz Index	12 (25.0%)	Bauco et al., 1996 [76]; Cheung & Lau, 2016 [43]; Lau & Cheung, 2016 [50]; Pedro et al., 2017 [55]; Ravaglia et al., 1997 [83]; Richmond et al., 2011 [90]; Schroyen et al., 2020 [57]	• Functional independence (ADLs): MED = 39.0% (Range : 19.0% – 60.8%)	**§**	**§**
		Bauco et al., 1996 [76]; Lau & Cheung, 2016 [50]; Ravaglia et al., 1997 [83]	• Bathing: MED = 65.1% (Range : 26.1% – 65.5%)	**§**	**§**
		Bauco et al., 1996 [76]; Infusino et al., 1996 [79]; Lau & Cheung, 2016 [50]; Ravaglia et al., 1997 [83]	Functional dependence (ADLs): • Dressing: MED = 46.8% (Range : 15.0% – 75.0%) • Using the toilet: MED = 40.0% (Range : 9.8% – 80.0%) • Transferring: MED = 37.6% (Range : 10.5% – 39.3%) • Incontinence: MED = 37.9% (Range : 28.1–75.0) • Eating: MED = 17.0% (Range : 2.6% – 50.0%)	**§**	**§**
		Cohen-Mansfield et al., 2013 [88]	**§**	ADL: M = 3.2	**ADL: 7 items;** • Rating: 4-point scale (0 = no difficulty to 3 = complete disability) • Score range: 0–21 • Interpretation: Higher scores indicated greater dependence.
		Tafaro et al., 2005 [84]	**§**	ADL: M = 2.5	• Scoring: unclear
		Dello Buono et al., 1998 [77]; Urciuoli et al., 1998 [86]^**†**^	**§**	ADL: MED of means = 12.1 (Range: 11.0% − 13.2%)	**ADL: 6 items;** • Rating: 3-point scale (1 = no difficulty to 3 = complete disability) • Score range: 6–18 • Interpretation: Higher scores indicated greater dependence.
		Lau & Cheung, 2016 [50]; Pedro et al., 2017 [55]	• Dependence in ≥ 1 ADL: MED = 57.4% (Range : 44.1% – 70.7%)	**§**	**§**
		Lau & Cheung, 2016 [50]	**§**	M = 0.9	• The mean score indicated the average number of dependent ADLs.
		Ravaglia et al., 1997 [83]	• Partial dependence in ADLs: 27.4%	**§**	**§**
		Dello Buono et al., 1998 [77]; Infusino et al., 1996 [79]; Ravaglia et al., 1997 [83]; Richmond et al., 2011 [90]	• Total dependence/Severe functional impairment in ADLs: MED = 30.3% (Range: 18.0% – 53.6%)	**§**	**§**
		Richmond et al., 2011 [90]	**§**	ADL: M = 3.7	**ADL: 6 items;** • Interpretation: ≤ 2 = Severe functional impairment 3 = Poor function 4 = Moderate impairment 5 = Good function 6 = Full function • Higher scores indicated greater independence.
Lawton Scale	11 (22.9%)	Cohen-Mansfield et al., 2013 [88]	**§**	IADL: M = 8.3	**IADL: 7 items;** • Rating: 4-point scale (0 = no difficulty to 3 = complete disability) • Score range: 0–21 • Interpretation: Higher scores indicated greater dependence.
		Dello Buono et al., 1998 [77]	• IADLs: 50% were fully dependent	**§**	**§**
		Dello Buono et al., 1998 [77]; Niimura et al., 2020 [53]; Urciuoli et al., 1998 [86]^**†**^	**§**	IADL: MED of means = 5.5 (Range: 3.2–6.9)	**IADL: 8 items;** • Rating: 2-point scale (0 = complete disability, 1 = no difficulty) • Score range: 0–8 • Interpretation: Higher scores indicated greater independence.
		Lau & Cheung, 2016 [50]	**§**	IADL: M = 2.1	• The mean score indicated the average number of dependent IADLs.
		Infusino et al., 1996 [79]	• IADLs: 27.5% were bed-confined and quite dependent for all the activities	**§**	**§**
		Madrigal-Leer et al., 2020 [52]	IADLs: • 63% were totally dependent • 12% were severely dependent • 16% were moderately dependent • 7% were mildly dependent • 2% were totally independent (35% were partially dependent)	**§**	**§**
		Lau & Cheung, 2016 [50]; Pedro et al., 2017 [55]	IADLs: • No dependence: MED = 18.9% (Range : 2.5% – 35.3%) • Dependence in ≥ 1 IADL: MED = 82.1% (Range : 66.7% – 97.4%)	**§**	**§**
		Lau & Cheung, 2016 [50]	IADLs dependence: • Shopping: 46.7% • Preparing meals: 41.8% • Washing clothes: 28.3% • Using public transport: 64.2% • Telephoning: 17.0% • Handling finances: 15.8%	**§**	**§**
		Song et al., 2022 [58]	IADL impairment: 64.7%	**§**	**§**
		Tindale et al., 2019 [62]	**§**	IADL: M = 15.6	**IADL: 8 items;** • Scoring: unclear • Max score: 23 • Interpretation: Higher scores indicated greater independence.
		Tafaro et al., 2005 [84]	**§**	IADL: M = 1.4	• Scoring: unclear
Barthel Index	10 (20.8%)	Li et al., 2022 [51]; Madrigal-Leer et al., 2020 [52]; Zhang et al., 2022 [66]	• Independence in ADLs: MED = 23.7% (Range: 5.0% – 32.2%)	**§**	**§**
		Feng et al., 2023 [46]; Li et al., 2022 [51]; Rabunal Rey et al., 2004 [82]; Sun et al., 2023 [60]; Yao et al., 2018 [64]; Zhang et al., 2022 [66]	• Dependence/impairment in ADLs: MED = 50.6% (Range: 36.6–76.3)	**§**	**§**
		Madrigal-Leer et al., 2020 [52]; Rabunal Rey et al., 2004 [82]	ADLs dependence levels: • Totally dependent: MED = 20.8% (Range: 18.5% – 23.0%) • Severely dependent: MED = 21.5% (Range: 13.0% – 30.0%) • Moderately dependent: MED = 23.1% (Range: 11.1% – 35.0%) • Mildly dependent: MED = 32.2% (Range: 7.0% – 57.4%)	**§**	**§**
		Molander et al., 2010 [80]	**§**	ADL: M = 13.1	**ADL: 10 items;** • Scoring varies: 0 to 1, 0 to 2, or 0 to 3 points, depending on the item • Score range: 0–20 • Interpretation: Higher scores indicated greater independence.
		Niimura et al., 2020 [53]	**§**	ADL: M = 80.9	**ADL: 10 items;** • Scoring varies: 0, 5, 10, or 15 points, depending on the item • Score range: 0–100 • Interpretation: Higher scores indicated greater independence.
		Yang, 2013 [89]	ADLs: • Score 0–10 (total dependence): 29.0% • Score 91–100 (total independence): 21.0%	**§**	**§**
The 13-question ADLs module	1 (2.1%)	Garasky et al., 2012 [71]	**§**	ADL: M = 25.5	**ADL: 13 items;** • Rating: 3-point scale (1 = disability to 3 = no difficulty) • Score range: 13–39 • Interpretation: Higher scores indicated greater functional ability.
Cumulative scale containing 5 personal ADLs and 4 IADLs^d^	1 (2.1%)	Nyqvist et al., 2006 [81]	**§**	ADL + IADL: M = 3.3	• Scores range from 0 (independent in all activities) to 9 (dependent in all activities). • The mean score indicated the average number of dependent activities.
6 major life ADLs^a^	1 (2.1%)	Ailshire et al., 2011 [67]	No ADL limitations: 18.0%	ADL: M = 2.4	**ADL: 6 items;** • Rating: 2-point scale (0 = no difficulty, 1 = complete disability) • Score range: 0–6 • Interpretation: Higher scores indicated greater dependence.
5 Functional Activities^b^	1 (2.1%)	Galenkamp et al., 2013 [78]	Functional abilities: • Moving indoors: 10.0% • Walking 400 m: 71.4% • Using stairs: 66.7% • Dressing/undressing: 19.0% Getting in/out of bed: 9.5%	**§**	**§**
5 ADLs^c^	1 (2.1%)	Herr et al., 2018 [48]	Disability for ≥ 2 ADL: 58.0%	**§**	**§**
DAFS-IADLs	1 (2.1%)	Margrett et al., 2010 [73]	**§**	IADL: M = 58.7	• Item and scoring details: unclear • Observed score range: 9–81 • Interpretation: Higher scores indicate better performance.
DAFS-R	1 (2.1%)	Davey et al., 2010 [70]	**§**	ADL: M = 16.48 IADL: M = 26.04	• Item and scoring details: unclear • Observed score range for ADLs: 0–23 • Observed score range for IADLs: 0–58
ADL-H	1 (2.1%)	Freeman et al., 2017 [47]	• Functional impairment in ADLs (ADL-H > 1): 81.7%	**§**	**§**
ADL-staircase	1 (2.1%)	von Heideken Wågert et al., 2006 [87]	ADLs independence: • Bathing: 20.0% • Continence, transfers, toileting, dressing: 40.0% to 60.0% • Eating: 80% IADLs independence: • Cleaning, grocery shopping, transport: <20.0% • Cooking: 20.0%	**§**	**§**
EQ-5D-3 L	1 (2.1%)	Chen et al., 2020 [42]	• Mobility: 42.6% had no problems; 52.9% had moderate problems; 4.6% had extreme problems. • Self-care: 59.8% had no problems; 36.0% had moderate problems; 4.2% had extreme problems.	**§**	**§**
Self-reported independence	1 (2.1%)	Wang et al., 2020 [63]	• Independence: 46.3% • Light independence: 32.5% • Moderate dependence: 16.3% • Severe dependence: 5.0%	**§**	**§**
Open question on functional challenges^e^	1 (2.1%)	Jopp et al., 2016 [49]	Main functional challenges: • Physical health/ADL restrictions: 42.7% • Mobility restrictions: 34.7%	ADL: M = 1.3	• The mean score indicated the average number of functional challenges reported by the participants.
Uclear^f^	2 (4.2%)	Deckers et al., 2018 [45]^**†**^	ADLs and IADLs: • No disability: 51.9% • Disability in only IADLs: 24.9% • Disability in both ADLs and IADLs: 23.3%	**§**	**§**
		Yang, 2013 [89]	IADLs: 3.0% were fairly independent for grocery shopping, meal preparation, house cleaning, laundry, taking medicine, telephoning, and handling finances.	**§**	**§**

*ADLs* (Personal/Basic) Activities of daily Living, *IADLs* Instrumental Activities of Daily Living, *ADL-H* Activities of Daily Living Hierarchy,* DAFS-IADLs* Direct Assessment of Functional Status-Instrumental Activities of Daily Living,* DAFS-R* Direct Assessment of Functional Status-Revised,* EQ-5D-3L* three-level EuroQol-5D scale, *OARS* Older Americans Resources and Services Multidimensional Functional Assessment Questionnaire

*Prevalence data: 'MED' represents the median prevalence rate for multiple studies using the same instrument. 'Range' indicates the range of prevalence rates for studies using the same instrument

**Score Data: 'M' denotes mean score. 'MED of Means' represents the median of mean scores across studies using the same instrument

§ Cells marked with this symbol indicate data that were either not reported by the authors or are not applicable

†Studies that described functional capacity in different subgroups (e.g., those living at home vs. in nursing homes). Combined mean prevalence rates and/or scores were computed for this table

^a^Walking across a room, dressing, bathing, eating, getting in and out of bed, and using the toilet

^b^Moving indoors, walking 400 m, using stairs, dressing and undressing, and getting in and out of bed

^c^Eating, dressing, getting in and out of bed, using the toilet, bathing or showering

^d^Bathing, dressing, going to the toilet, transfer, feeding, cleaning, food shopping, transportation and cooking

^e^ *“Now please think for a moment about the things that you find challenging. Are there things that you find challenging or difficult?”*

^f^The instruments used for assessment were not presented by the authors

In terms of ‘functional dependence’ in IADLs, the reported prevalence rates also varied significantly. One study documented a rate of 64.7% [58] and another indicated that a mere 3% of its participants were ‘fairly independent’ in performing IADLs, thus suggesting that 97% had some level of dependence [89]. The median prevalence of 82.1% was computed from two grouped studies using the same instrument [50, 55]. For ‘total functional dependence’ in IADLs—indicating complete reliance on others for all IADLs—reported prevalence rates were 27.5% [79], 50% [77] and 63% [52] (Table 3). For a visual representation of the prevalence of functional dependence in one or more ADLs or IADLs, refer to Additional file 8.

Several studies reported mean scores. As illustrated in Table 3, there was a notable variation in scoring strategies, even when employing the same instrument. For instance, while some studies using the OARS clearly differentiated between ADLs and IADLs, each with its own set of items and scoring range, others combined them. Moreover, the interpretation of scores—whether a higher score indicated increased independence or dependence—also varied between studies. Still focusing on the OARS—often used to assess both ADL and IADL domains—the computed median of mean scores, derived from grouped studies, was 9.6 points for ADLs [12, 39–41, 61, 69, 70] and 5.7 points for IADLs [39–41, 61, 65, 69, 70]. This suggests a heightened level of functional dependence in the IADLs domain (considering the scoring range of 0–14, where a higher value indicated more independence). In summary, the documented mean scores reflecting participants’ ability to carry out ADLs and IADLs showed considerable variability across studies, from mild to severe levels of dependence, as detailed in Table 3.

Lastly, some of the studies retained differentiated between general and specific dependence on targeted ADLs and IADLs (e.g., bathing, dressing, toileting, housework, grocery shopping, cooking) [12, 50, 74, 76, 78, 79, 83, 87, 89] (Table 3).

A total of 16 instruments (or approaches) for assessing functional dependence were identified across the included studies and are presented in Table 3, which lists the instruments reported in 48 of the 53 included studies. Although five studies [53, 55, 58, 74, 84] were excluded from the Table due to the absence of specific prevalence data on functional dependence, they were incorporated into the review for their investigation into the associations between depressive symptoms and functional dependence. Notably, two instruments stood out in popularity: the OARS Multidimensional Functional Assessment Questionnaire and the Katz Index. Each was employed in 12 (25.0%) of the reviewed studies. In some studies (*n* = 9, 18.8%), researchers opted to combine two screening instruments (Katz Index and Lawton Scale or Barthel Index and Lawton Scale) to cover both domains of functional health, i.e., ADLs and IADLs [50, 52, 53, 55, 77, 79, 84, 86, 88]. For a visual representation of the identified instruments to assess functional dependence, see Additional file 9.

A marked heterogeneity characterised the representation of functional dependence across the studies. Terminology ranged from overall ‘functional dependence’ to ‘disability’, as well as categorical classifications such as ‘mild,’ ‘moderate,’ or ‘severe’ dependence. Furthermore, some studies detailed their findings by associating levels of dependence with specific activities. Assessments also varied in their focus—while some exclusively evaluated ADLs (e.g [43, 47, 51]), others were centered on IADLs (e.g [58, 65, 73]), and several addressed both domains (e.g [12, 40, 52]). This variability necessitated a segmented analysis approach to ensure accurate representation and avoid potential aggregation of differing outcomes. For a detailed overview of the prevalence rates and mean scores by instrument used, see Table 3.

### Associations between depressive symptoms and functional dependence

Our fourth research question aimed to determine whether studies documented associations between depressive symptoms and functional dependence in near-centenarians and centenarians. Table 4 provides detailed information from the studies specifically addressing these associations.

**Table 4:** Relationship between depressive symptoms and functional dependence (*n* = 16)

Studies (1st author and year of publication)	Correlation/Relationship	Statistical significance	Additional notes/findings
Cheung & Lau, 2016 [43]	*r* = −0.02	*p* > 0.05	ADL and GDS scores were not correlated.
Cimarolli & Jopp, 2014 [68]	*r* = 0.47	*p* < 0.01	Higher depressive symptomatology contributed to 4.9% of variance in functional disability.
Cimarolli et al., 2018 [44]	*r* = 0.48	*p* < 0.01	Depressive symptoms were significantly associated with higher functional disability. Functional disability explained 4.0% of the variance in predicting depressive symptoms.
Jopp et al., 2016 [49]	*r* = 0.11	*p* > 0.05	No significant association between functional challenges and depression.
Jopp, Park et al., 2016 [12]	*r* = −0.37 (ADL) *r* = −0.38 (IADL)	*p* < 0.05	GDS scores were negatively correlated with ADL and IADL scores. IADL dependence was a significant predictor for depression, explaining 7.1% of variance.
Margrett et al., 2010 [73]	Not exclusively specified for the target variables.	N/A	Functional indicators played a minor role in mental health outcomes. Objective ADL ratings were not significant predictors of depressive symptoms.
Martin et al., 2000 [75]	*β* = 0.14 (ADL) *β* = −0.21 (IADLs)	*p* > 0.05	ADLs and IADLs were not predictors of depression.
Oseland et al., 2016 [54]	*r* = −0.37	*p* ≤ 0.001	Bivariate correlation observed between functional health and depressive symptoms.
Ravaglia et al., 1997 [83]	*r*_*men*_ = −0.14 *r*_*women*_ = −0.54	*p*_*men*_ = 0.404 *p*_*women*_ = 0.001	*For women*: Depressive symptoms and functional dependence showed a strong negative correlation. Functional dependence (ADL) was a significant predictor for depressive symptoms (GDS), explaining 28.0% of variance. Depressive symptoms (GDS) were a significant predictor for functional dependence (ADL), explaining 27.0% of variance. *For men*: Correlation was weak and not statistically significant.
Ribeiro et al., 2018 [56]	OR = 0.82	*p* = 0.002	Dependence in ADLs was a predictive factor for depression, with higher ADL scores (indicating better functionality) being associated with lower odds of depression
Richmond et al., 2011 [90]	*F* (1.145) = 2.30	*p* = 0.13	No significant association observed between depression and physical functioning.
Song et al., 2022 [58]	χ² = 26.30	*p* < 0.05	Higher depression prevalence observed in centenarians with impaired daily activity functioning (34.0% vs. 18.6%).
Struckmeyer et al., 2021 [59]	*r* : −0.37	*p* < 0.01	Negative correlation observed between functional health and depressive affect.
Sun et al., 2023 [60]	OR = 2.05	*p* < 0.05	Lower physical disability was associated with higher odds of depressive disorder. Higher physical disability observed in centenarians with depressive disorder (47.4% vs. 32.8%).
Tafaro et al., 2002 [85]	*r* = 0.06	*p* = 0.4	No correlation between GDS scores and IADL.
Yao et al., 2018 [64]	*β* = 0.94, OR = 2.57	*p* < 0.001	ADL impairments were linked to higher odds of depression. Higher ADL impairment observed in centenarians with depression (72.3% vs. 48.8%.)

*ADL* Activities of Daily Living,* IADL* Instrumental Activities of Daily Living

Note 1: for specific measures of depressive symptoms and functional dependence, refer to tables 2 and 3

Note 2: *r* = Pearson correlation coefficient; *p* = p-value indicating significance; *β* = standardized regression coefficient; OR = Odds Ratio; *F* = F-statistic from an ANOVA; χ² = ki-squared

From the 53 studies included in this scoping review, 16 (30.2%) explored the above-mentioned relationship in this specific age group. Five (31.3%) of these studies found no significant correlation between these conditions [43, 49, 73, 75, 85]. Conversely, nine (56.3%) identified significant relationships between depressive symptoms and functional dependence [12, 44, 54, 56, 58–60, 68, 83]. Additionally, two (12.5%) studies observed a higher prevalence of depressive symptoms among those participants showing dependence in IADLs or vice-versa [12, 58] (Table 4).

Moreover, six (37.5%) studies investigated depressive symptoms as a potential predictor of functional dependence or the inverse [12, 44, 56, 60, 64, 83]. Of these, four (66.7%) indicated that functional dependence might be a predictive factor for depressive symptoms or vice versa [12, 44, 56, 83], while two (33.3%) did not report significant relationships [12, 75] (Table 4).

## Discussion

This scoping review aimed to comprehensively map and summarize the available studies on depressive symptoms and functional dependence in near-centenarians and centenarians. Overall, our main results showed substantial variability in prevalence rates for these conditions and suggested a significant association between them.

### Geographic diversity and research gaps

The research landscape demonstrated geographic and cultural diversity, with studies originating from North America and Europe, each accounting for over one third of the studies, and Asia, accounting for one fourth of the studies. While a notable number of studies came from the USA, China, and Italy, a significant research gap became evident for studies originating from Africa and South America, represented in none of the studies, and Australia, represented in one study. The WHO [91] reports a healthy life expectancy at birth in Africa of 56 years in 2019, a stark contrast to the European Union’s 64.5 years for women and 63.5 years for men in 2020, providing context to this research void [92, 93]. Concurrently, in 2019, South America displayed a varied range of average healthy life expectancy at birth, from 57.2 years in Guyana to 70 years in Chile. The relative lack of centenarian-focused studies from these regions could be attributed to multifaceted factors, including economic factors, healthcare infrastructure, and prevailing research priorities, rather than the disparities in life expectancy alone. The USA consistently emerged as a significant contributor to the number of retained studies as previously mentioned. This fact can be attributed to the country’s large population of centenarians, which likely influences the greater volume of research originating from the region. Additionally, Italy’s frequent representation in European studies might relate to its high ratios of centenarians and the recognized ‘blue zone’ status of specific regions [94].

### Challenges in centenarian mental health research

While research interest in centenarians’ mental health and its functional repercussions spans across continents, the volume of published research remains somewhat limited. A previous systematic review has acknowledged a scarcity of literature focusing on centenarian’s mental health [13]. Potential challenges, such as recruiting very old individuals, especially those with cognitive limitations [95], combined with societal obstacles like ageism, negative stereotypes, and logistical challenges [96, 97], may account for this research disparity. This gap is particularly concerning given the significant role older adults play as major consumers of healthcare services [95, 97, 98].

### Methodological approaches and research design

While our review highlights the use of quantitative methodologies in studying depressive symptoms among centenarians, incorporating qualitative approaches, such as phenomenological designs, could uncover nuances specific to this age group. Qualitative methods can reveal subtler, age-specific aspects of depression, offering insights into experiences that differ from younger populations [99]. The integration of these approaches in future research on centenarians, particularly through mixed-methods designs [100], could offer a more comprehensive understanding of depressive symptomatology in very old age. The predominant use of cross-sectional designs in the reviewed studies highlights their utility in examining variable relationships and prevalence rates in this niche field [99]. Yet, there is a notable absence of longitudinal research on centenarians’ mental and functional health trajectories [101]. As health conditions can undergo rapid changes in advanced age [102, 103], longitudinal studies could offer critical insights not only into incidence rates, health fluctuations, and even determinants of longevity [104], but also in elucidating the evolving relationship between depressive symptoms and functional dependence over time.

### Prevalence of depressive symptoms and functional dependence

Our scoping review underscored a wide range in depressive symptom prevalence with individually reported study rates ranging from approximately 10.5% to 73%. Notably, when considering the mean prevalence rates derived from grouped studies using similar instruments, this range narrows to approximately 12.7% to 27.1%. This finding suggests that employing consistent screening tools across studies can lead to more reliable and comparable results, despite differences in samples and cultural contexts. This consistency is crucial in accurately understanding and addressing depressive symptoms in very old age, reinforcing the need for standardized approaches in mental health research. This aligns with Luppa et al.‘s findings [105], who observed prevalence rates spanning from 4.5% to 37.4% in older adults aged ≥ 75 years. The mean scores from various screening instruments highlighted in our findings corroborate the idea that centenarians do not suffer from depressive symptoms more often than their ‘younger’ older adult counterparts. Despite the considerable variations in prevalence across studies, our findings clearly indicate that depressive symptoms remain a prevalent problem in very old age, and deserving of investment in their prevention, treatment, and support.

In parallel, our results also showed a meaningful range of prevalence rates for functional dependence among the targeted population. For ADLs, rates varied from about 58% to 82%, with median prevalence in grouped studies between 50.6% and 57.4%. In IADLs, rates were as high as 97%, with a median of 82.1% in grouped studies. This highlights the significant extent of functional dependence in this age group, underscoring the need for tailored care interventions that are informed by a clear understanding of their everyday care needs. Centenarians exhibited higher levels of functional dependence than their ‘younger’ older adult counterparts—a difference likely attributable to the greater frailty typically observed in centenarian populations [48, 106, 107]. A systematic review focusing on older Asian adults reported pooled prevalence rates of 21.5% for ADLs and 46.8% for IADLs [108], further supporting this observation.

Hence, a notable aspect of this scoping review is the considerable variability found across studies when examining depressive symptoms and functional dependence in near-centenarians and centenarians. While the use of different measures or instruments could play a role, these differences could further arise from varying methodological approaches, such as sampling techniques (e.g., degree of representativeness of the sample). Cultural contexts might also contribute to differences in prevalences across studies. For instance, variations in cultural norms and values surrounding mental and functional health in aging can result in divergent perceptions and reporting of depressive symptoms and functional dependence.

### Screening for depressive symptoms

A diverse array of instruments has been employed to screen for depressive symptoms. However, the Geriatric Depression Scale (GDS) was the predominant instrument used in the retained studies, aligning with existing literature; Cheng et al. [13] noted that the GDS-30 and the GDS-15 have been frequently employed in research involving near-centenarians and centenarians. The popularity of the GDS, specifically designed for geriatric populations [109], is likely attributable to its suitability for very old individuals. Balsamo et al. [110] suggested the GDS-30 might be the most effective self-assessment tool for screening depressive symptoms in this age group. Globally, self-assessment instruments dominate the landscape and serve as valuable tools for both clinical and research purposes in evaluating depressive symptoms’ presence and severity. However, for individuals with major neurocognitive impairment, self-assessments may be less reliable. Our review, supported by findings from Cheng et al. [13], highlights a notable lack of utilization of hetero-assessment instruments, such as the Cornell Scale for Depression in Dementia (CSDD), among centenarians. As the global population continues to age, and consequently, the number of cases of neurocognitive disorders increases [111], the consideration of hetero-assessment instruments becomes vital for those suffering from this type of condition. While the DSM and the ICD criteria are gold-standard for the assessment of depressive symptoms, their rigorous application often demands significant resources [13], which explains their absence in the studies we reviewed. Notably, both the ICD and DSM criteria do not cater specifically to older adults. Furthermore, to our knowledge, no tools have been validated specifically to screen depressive symptoms in centenarians.

### Screening for functional dependence

Multiple instruments have been employed to screen for functional dependence. The OARS Multidimensional Functional Assessment Questionnaire [112] and the Katz Index [32, 33] emerged as predominant, and the consistent usage of these instruments underscores their importance in the field. Others, like the Lawton Scale [34] and the Barthel Index [113], were also commonly employed, albeit to a lesser extent. Interestingly, despite the broad application of these tools, the current literature seems to lack universal guidelines that recommend the best methods for measuring functional dependence in centenarians. Nevertheless, originating as a tool for program evaluation and resource allocation, the OARS, with its ADL and IADL subscales, has found consistent application in populations of older adults, including centenarians [40, 56, 59]. Moreover, the Katz Index remains one of the most effective instruments for assessing older adults’ capacity to perform the basic ADLs. Thus, despite some limitations, such as its restricted ability to measure small changes, this instrument is still used extensively in diverse care settings. Its use has contributed significantly to establishing a common language when discussing functional health internationally [114], which is particularly relevant in the current context of rapid demographic ageing world-wide. It should be noted, however, that it is beneficial to pair the Katz Index with another instrument assessing IADLs for a comprehensive evaluation, as observed in certain analyzed studies.

### Association between depressive symptoms and functional dependence

Our review highlights a potential link between depressive symptoms and functional dependence in near-centenarians and centenarians, indicating an association worth further investigation. Research on community-dwelling, cognitively healthy older adults has shown that functional dependence can amplify depressive symptoms, likely due to a diminished sense of purpose and weakened resilience [115]. In line with this, a subset of the studies we reviewed revealed a predominantly moderate yet significant association between depressive symptoms and functional dependence. These findings suggest the bidirectional nature of this relationship: not only can depressive symptoms heighten the risk of functional dependence, but the onset of functional dependence can also aggravate depressive symptoms.

Given enhanced physical and social vulnerability in very old age, understanding this interconnected relationship becomes a priority. Breaking this potentially adverse cycle could interrupt the cascade of undesirable outcomes that might emerge as a consequence. Interestingly, functional impairments seem to be more pronounced among centenarians, but this does not necessarily translate to a higher prevalence of depressive symptoms compared to their ‘younger’, mostly healthier, older counterparts.

### Interpreting depressive symptom prevalence in centenarians: resilience and age-related biological changes

Building on the previous observation, the concept of resilience among centenarians becomes particularly interesting. Some authors suggested that while ‘younger’ older adults’ emotional well-being may be more adversely affected by functional disabilities, centenarians appear more resilient to these negative influences [72, 116]. This resilience, as a vital health determinant, is observed in greater instances among centenarians, suggesting they retain this protective trait longer than ‘younger’ older adults [117]. This resilience might serve as a potential shield against the adverse effects of functional dependence and is worthy of further exploration.

At the same time, a gerontobiological perspective invites a more nuanced interpretation of depressive symptomatology in very old age. The literature indicates that age-related reductions in key neurotransmitters—such as serotonin and dopamine—can affect emotional regulation and impact the clinical expression of mood disorders. Likewise, neuronal loss and structural atrophy within fronto-limbic circuits play a central role in affect regulation, which may worsen depressive symptomatology or contribute to atypical manifestations in this age group (e.g., less sadness and more apathy) [118–121]. Furthermore, the hypothesis of chronic low-grade inflammation associated with ageing (inflammaging, and more specifically neuroinflammaging) suggests cumulative effects on cerebral tissues and has been linked to depression [122–124]. Similarly, both late-life depression and its treatment resistance are likely to be associated with vascular factors [125, 126] and possibly amyloid pathology [127]. These biological changes represent an additional element that should be considered when assessing depression among the oldest old.

Ultimately, while centenarians may indeed possess unique protective factors, the reported rates of depressive symptoms and functional dependence in this unique population are by no means negligible. Their potential vulnerability—whether psychosocial or biological—underscores the need to strengthen protective mechanisms and to optimize prevention, detection, and evidence-based treatment strategies.

A summary of the main research gaps and directions for future research is available in Additional file 10.

### Limitations

This scoping review presents some limitations. First, although a comprehensive and rigorous search strategy was implemented, this work remains subject to the inherent limitations of literature reviews, including the possibility that some relevant references may not have been identified during the study selection process, which was nevertheless conducted through independent title and abstract screening by two reviewers. Second, we excluded two studies written in Chinese due to their non-translatable format, potentially sidelining valuable insights they might have offered. Third, potential biases in the findings of the included studies cannot be ruled out. Given that scoping reviews do not necessitate a qualitative evaluation of the sources, we did not appraise the methodological quality of the incorporated evidence. Consequently, we cannot provide graded recommendations based on this review. Fourth, another limitation of our review is the inclusion of papers derived from the same larger studies, resulting in possible non-independent samples. This overlap might have amplified certain findings while underrepresenting others, particularly given the smaller overall number of studies included. Fifth, given the substantial variability in the instruments and approaches used to assess depressive symptoms and functional dependence—as well as differences in scoring and interpretation across the included studies—we cannot guarantee that our synthesis is fully aligned with the original authors’ perspectives.

## Conclusions

This scoping review, spanning diverse geographies and cultures, examined the existing scientific literature on depressive symptoms and functional dependence in near-centenarians and centenarians. It revealed geographical disparities, as well as a notitceable absence of studies from Africa and South America, and a scarcity in qualitative and mixed-methods research published on the topic. The predominance of quantitative, cross-sectional studies underscores the need for more diversified and longitudinal research approaches to better understand the complex health trajectories and dynamic needs of this age group.

Our review spotlighted significant variability in the instruments used, with self-assessment tools like the GDS predominating in screening for depressive symptoms, despite their potential limitations in individuals with neurocognitive impairment. Concurrently, a variety of instruments, including the OARS and the Katz Index, were deployed to assess functional dependence. The employment of diverse scoring strategies and thresholds in various studies complicates the synthesis of reported findings, posing challenges in deriving clear and consistent conclusions regarding the functional status of this age group. These findings underscore the need for the development of harmonised approaches to ensure the comparability of results across regions within this unique population.

A subset of the reviewed studies elucidated the reciprocal relationship between depressive symptoms and functional dependence, highlighting their mutual influence and potential to escalate adverse outcomes. Our findings underscored that, despite higher levels of functional dependence, centenarians do not necessarily show higher depressive symptoms when compared to their ‘younger’ older counterparts. This suggests the presence of underlying mechanisms, such as resilience, that potentially mitigate the negative implications of functional impairments, marking an important avenue for future studies.

Nevertheless, the significant prevalence of depressive symptoms and functional dependence among near-centenarians and centenarians calls for thoughtful attention, preventive measures, and effective, evidence-based interventions. Such interventions should address functional challenges and alleviate depressive symptoms, ultimately improving overall well-being and quality of life in this very old age group.

### Implications for research

The diversification of research methodologies, including longitudinal and qualitative studies, would play a key role in gaining deeper insights into the needs and health trajectories of near-centenarians and centenarians. In addition, broadening the geographical scope would further contribute to a comprehensive understanding of diverse cultures and environmental contexts.

Moreover, establishing clear guidelines for frequently employed instruments, or developing refined alternatives, offers a noteworthy avenue for further investigation. Standardizing scoring strategies and thresholds will facilitate the synthesis of heterogeneous data, enabling more consistent conclusions and reliable international comparisons regarding depressive symptoms and functional dependence in this population.

Thorough study of the link between depressive symptoms and functional dependence is essential for crafting targeted interventions. In parallel, reaching a consensus on criteria for identifying depressive symptoms and signs in very old individuals holds significant importance. A recent e-Delphi study [128] represents an initial step toward improving depression assessment in near-centenarians and centenarians. The findings suggest that developing and testing a more adapted screening tool could enhance early detection and intervention, a direction that merits further consideration in future research.

### Implications for practice

The development and implementation of multifaceted, scientifically grounded interventions are necessary to address the prevalent depressive symptoms and functional dependence in this age group. Proactive preventive measures and early identification and management of symptoms and signs are imperative to mitigate the impact of depressive symptomatology on functional decline, or vice-versa. Enhancement of professional competency, caregiver support, and educational initiatives can foster age-sensitive care, reduce attitudes of ageism and promote mental and functional well-being. Advocacy for informed policies, enhancement of healthcare infrastructure, and resource allocation are essential to address the unique needs of near-centenarians and centenarians, both in terms of their mental and functional health. Promoting public awareness and engagement is essential to fostering societal inclusion, mitigating stigma, and reinforcing community support for this age group.

## Supplementary Information

Additional file 1: Bibliographic database search strategies.

Additional file 2: Sources excluded following a full-text review.

Additional file 3: Data extraction instrument.

Additional file 4: Average number of studies by 5-year publication periods.

Additional file 5: Number of studies per country.

Additional file 6: Visual representation of the prevalence of depressive symptoms.

Additional file 7: Identified instruments to screen for depressive symptoms.

Additional file 8: Visual representation of the prevalence of functional dependence.

Additional file 9: Identified instruments to assess functional dependence.

Additional file 10: Summary of the main research gaps and future directions.

## Data Availability

No datasets were generated or analysed during the current study.

## Abbreviations

ADLs: Activities of Daily Living
APA: American Psychological Association
CINAHL: Cumulative Index to Nursing and Allied Health Literature
CSDD: Cornell Scale for Depression in Dementia
DSM: Diagnostic and Statistical Manual of Mental Disorders
GDS: Geriatric Depression Scale
IADLs: Instrumental Activities of Daily Living
ICD: International Classification of Diseases
JBI: Joanna Briggs Institute
OARS: Older Americans Resources and Services Multidimensional Functional Assessment Questionnaire
PQDTGlobal: ProQuest Dissertations & Theses Global
PRISMA-ScR: Preferred Reporting Items for Systematic Reviews and Meta-Analyses Extension for Scoping Reviews
USA: United States of America
WHO: World Health Organization
