# Supplementary material for: Depressive symptoms and functional dependence in near-centenarians and centenarians: a scoping review
Source: BMC Geriatr. 2026 Feb 6;26:321. doi: 10.1186/s12877-026-07026-4 (PMC12977654; doi:10.1186/s12877-026-07026-4)
Supplement: Supplementary file 1 — Additional file 1: Bibliographic database search strategies. [file 12877_2026_7026_MOESM1_ESM.docx]

**Additional file 1.** Bibliographic database search strategies

Depressive symptoms and functional dependence in near-centenarians and centenarians: a scoping review / Carla Gomes da Rocha, Armin von Gunten, Joëlle Rosselet Amoussou, Sofia Fernandes, Kim Uittenhove, Daniela S. Jopp, Olga Ribeiro and Henk Verloo

**Bibliographic database search strategies**

The search strategies were peer-reviewed by a second information specialist prior to execution.

**Embase.com**

1246 references found, 28 August 2023

("90 year*" OR "95 year*" OR nonagenarian* OR centenarian* OR supercentenarian* OR "aged 100" OR "100 year*" OR "oldest old*" OR "very old*" OR super-senior*):ab,ti,kw AND ('depression'/exp OR 'depression assessment'/exp OR (depress* OR "mood decline"):ab,ti,kw) AND ('daily life activity'/exp OR 'independent living'/exp OR 'health status'/exp OR 'walking difficulty'/exp OR 'ADL disability'/exp OR 'locomotion'/de OR 'walking'/exp OR 'amputee'/exp OR 'personal autonomy'/de OR 'quality of life'/exp OR 'functional disease'/exp OR 'physical disability'/exp OR 'community living'/exp OR 'physically disabled person'/exp OR ("daily living" OR "daily activit*" OR "daily life" OR "everyday functioning" OR "limitation of activit*" OR independen* OR autonomy OR "aging in place" OR ((physical* OR functional) NEXT/4 (health OR decline OR disab* OR frailty OR activit* OR inactivit* OR parameter* OR impair* OR condition* OR status OR abilit* OR limit* OR incapacit* OR capacit* OR dependenc* OR performance* OR function* OR deficien*)) OR (functional NEXT/3 disorder*) OR mobility OR ambulation OR walking OR locomotion OR "quality of life" OR "life quality" OR "self-rated health"):ab,ti,kw)

**Medline ALL Ovid**

Ovid MEDLINE(R) ALL 1946 to August 25, 2023

928 references found, 28 August 2023

(Centenarians/ OR Nonagenarians/ OR ("90 year*" OR "95 year*" OR nonagenarian* OR centenarian* OR supercentenarian* OR "aged 100" OR "100 year*" OR "oldest old*" OR "very old*" OR super-senior*).ab,ti,kf.) AND (Depression/ OR exp Depressive Disorder/ OR Patient Health Questionnaire/ OR (depress* OR "mood decline").ab,ti,kf.) AND ("Activities of Daily Living"/ OR "Independent Living"/ OR exp "Health Status"/ OR "Functional Status"/ OR "Quality of Life"/ OR "Mobility Limitation"/ OR "Locomotion"/ OR exp "Walking"/ OR disabled persons/ OR amputees/ OR persons with hearing impairments/ OR visually impaired persons/ OR ("daily living" OR "daily activit*" OR "daily life" OR "everyday functioning" OR "limitation of activit*" OR independen* OR autonomy OR "aging in place" OR ((physical* OR functional) ADJ4 (health OR decline OR disab* OR frailty OR activit* OR inactivit* OR parameter* OR impair* OR condition* OR status OR abilit* OR limit* OR incapacit* OR capacit* OR dependenc* OR performance* OR function* OR deficien*)) OR (functional ADJ3 disorder*) OR mobility OR ambulation OR walking OR locomotion OR "quality of life" OR "life quality" OR "self-rated health").ab,ti,kf.)

**CINAHL with Full Text**

399 references found, 28 August 2023

(MH "Centenarians" OR MH "Nonagenarians" OR TI ("90 year*" OR "95 year*" OR nonagenarian* OR centenarian* OR supercentenarian* OR "aged 100" OR "100 year*" OR "oldest old*" OR "very old*" OR super-senior*) OR AB ("90 year*" OR "95 year*" OR nonagenarian* OR centenarian* OR supercentenarian* OR "aged 100" OR "100 year*" OR "oldest old" OR "very old*" OR super-senior*)) AND (MH "Depression+" OR MH "Geriatric Depression Scale" OR TI (depress* OR "mood decline") OR AB (depress* OR "mood decline")) AND (MH "Activities of Daily Living+" OR MH "Quality of Life+" OR MH "Community Living+" OR MH "Health Status+" OR MH "Functional Status" OR MH "Locomotion+" OR MH "Persons with Disabilities" OR MH "Amputees" OR TI ("daily living" OR "daily activit*" OR "daily life" OR "everyday functioning" OR "limitation of activit*" OR independen* OR autonomy OR "aging in place" OR ((physical* OR functional) W3 (health OR decline OR disab* OR frailty OR activit* OR inactivit* OR parameter* OR impair* OR condition* OR status OR abilit* OR limit* OR incapacit* OR capacit* OR dependenc* OR performance* OR function* OR deficien*)) OR (functional W2 disorder*) OR mobility OR ambulation OR walking OR locomotion OR "quality of life" OR "life quality" OR "self-rated health") OR AB ("daily living" OR "daily activit*" OR "daily life" OR "everyday functioning" OR "limitation of activit*" OR independen* OR autonomy OR "aging in place" OR ((physical* OR functional) W3 (health OR decline OR disab* OR frailty OR activit* OR inactivit* OR parameter* OR impair* OR condition* OR status OR abilit* OR limit* OR incapacit* OR capacit* OR dependenc* OR performance* OR function* OR deficien*)) OR (functional W2 disorder*) OR mobility OR ambulation OR walking OR locomotion OR "quality of life" OR "life quality" OR "self-rated health"))

**APA PsycInfo Ovid**

APA PsycInfo 1806 to August Week 3 2023

639 references found, 28 August 2023

("90 year*" OR "95 year*" OR nonagenarian* OR centenarian* OR supercentenarian* OR "aged 100" OR "100 year*" OR "oldest old*" OR "very old*" OR super-senior*).mp. AND (exp affective disorders/ OR "depression (emotion)"/ OR (depress* OR "mood decline").mp.) AND (exp self-care skills/ OR exp health status/ OR functional status/ OR physical mobility/ OR locomotion/ OR walking/ OR gait/ OR physical disorders/ OR ("daily living" OR "daily activit*" OR "daily life" OR "everyday functioning" OR "limitation of activit*" OR independen* OR autonomy OR "aging in place" OR ((physical* OR functional) ADJ4 (health OR decline OR disab* OR frailty OR activit* OR inactivit* OR parameter* OR impair* OR condition* OR status OR abilit* OR limit* OR incapacit* OR capacit* OR dependenc* OR performance* OR function* OR deficien*)) OR (functional ADJ3 disorder*) OR mobility OR ambulation OR walking OR locomotion OR "quality of life" OR "life quality" OR "self-rated health").mp.)

**Web of Science Core Collection**

Science Citation Index Expanded (1900-present), Social Sciences Citation Index (1900-present), Arts & Humanities Citation Index (1975-present), Conference Proceedings Citation Index-Science (1990-present), Book Citation Index (2005-present), Emerging Sources Citation Index (2005-present), Current Chemical Reactions and Index Chemicus

Advanced search > More options > Exact search

972 references found, 28 August 2023

TS=(("90 year*" OR "95 year*" OR nonagenarian* OR centenarian* OR supercentenarian* OR "aged 100" OR "100 year*" OR "oldest old*" OR "very old*" OR super-senior*) AND (depress* OR "mood decline") AND ("daily living" OR "daily activit*" OR "daily life" OR "everyday functioning" OR "limitation of activit*" OR independen* OR "autonomy" OR "aging in place" OR ((physical* OR "functional") NEAR/3 ("health" OR "decline" OR disab* OR "frailty" OR activit* OR inactivit* OR parameter* OR impair* OR condition* OR "status" OR abilit* OR limit* OR incapacit* OR capacit* OR dependenc* OR performance* OR function* OR deficien* OR "status")) OR ("functional" NEAR/2 disorder*) OR "mobility" OR "ambulation" OR "walking" OR "locomotion" OR "quality of life" OR "life quality" OR "self-rated health"))

**Cochrane Database of Systematic Reviews Wiley**

Issue 8 of 12, August 2023

0 references found, 28 August 2023

**(**((90 NEXT year*) OR (95 NEXT year*) OR nonagenarian* OR centenarian* OR supercentenarian* OR "aged 100" OR (100 NEXT year*) OR (oldest NEXT old*) OR (very NEXT old*) OR (super NEXT senior*)):ab,ti,kw AND (depress* OR "mood decline"):ab,ti,kw AND ("daily living" OR (daily NEXT activit*) OR "daily life" OR "everyday functioning" OR (limitation NEXT activit*) OR independen* OR autonomy OR "aging in place" OR ((physical* OR functional) NEXT/4 (health OR decline OR disab* OR frailty OR activit* OR inactivit* OR parameter* OR impair* OR condition* OR status OR abilit* OR limit* OR incapacit* OR capacit* OR dependenc* OR performance* OR function* OR deficien*)) OR (functional NEXT/3 disorder*) OR mobility OR ambulation OR walking OR locomotion OR "quality of life" OR "life quality" OR "self rated health"):ab,ti,kw**)**

**Cochrane Central Register of Controlled Trials Wiley**

Issue 7 of 12, July 2023

123 references found, 28 August 2023

**(**((90 NEXT year*) OR (95 NEXT year*) OR nonagenarian* OR centenarian* OR supercentenarian* OR "aged 100" OR (100 NEXT year*) OR (oldest NEXT old*) OR (very NEXT old*) OR (super NEXT senior*)):ab,ti,kw AND (depress* OR "mood decline"):ab,ti,kw AND ("daily living" OR (daily NEXT activit*) OR "daily life" OR "everyday functioning" OR (limitation NEXT activit*) OR independen* OR autonomy OR "aging in place" OR ((physical* OR functional) NEXT/4 (health OR decline OR disab* OR frailty OR activit* OR inactivit* OR parameter* OR impair* OR condition* OR status OR abilit* OR limit* OR incapacit* OR capacit* OR dependenc* OR performance* OR function* OR deficien*)) OR (functional NEXT/3 disorder*) OR mobility OR ambulation OR walking OR locomotion OR "quality of life" OR "life quality" OR "self rated health"):ab,ti,kw**)**

Additional searches

**Google Scholar**

<https://scholar.google.com/>

200 references consulted, February 11, 2021

Centenarian depression|"mood decline" "functional dependence"|"functional status"

**Dart Europe**

<https://www.dart-europe.org/basic-search.php>

8 references found, January 28, 2021

(centenarian* OR supercentenarian*) AND (functional OR functioning)

**ProQuest Dissertations & Theses Global (PQDTGlobal)**

<https://www.proquest.com/pqdtglobal>

Search options :

- Doctoral dissertations only
- NOFT - Anywhere except full text

(centenarian* OR supercentenarian* OR super-centenarian* OR near-centenarian* OR super-senior*) AND (functional OR functioning)

18 references found, January 28, 2021

(centenarian* OR supercentenarian* OR super-centenarian* OR near-centenarian* OR super-senior*) AND (depressive OR depression* OR mood)

6 references found, January 28, 2021

**Open Grey**

<http://www.opengrey.eu/>

20 references found, January 29, 2021

centenarian* OR centenaire*
