# Supplementary material for: Depressive symptoms and functional dependence in near-centenarians and centenarians: a scoping review
Source: BMC Geriatr. 2026 Feb 6;26:321. doi: 10.1186/s12877-026-07026-4 (PMC12977654; doi:10.1186/s12877-026-07026-4)
Supplement: Supplementary file 2 — Additional file 2: Sources excluded following a full-text review. [file 12877_2026_7026_MOESM2_ESM.docx]

**Additional file 2.** Sources excluded following a full-text review

Abril Chambo VJ, Musitu Ochoa G. A community study on the health of high risk populations: Social, physical and psychological determinants. *Revista de Psicologia Social Aplicada.* 2000;10(2):1-25.

***Reason for exclusion:*** Did not meet the inclusion criteria (population).

Aftab A, Lam JA, Thomas ML, Daly R, Lee EE, Jeste DV. Subjective age and its relationships with physical, mental, and cognitive functioning: A cross-sectional study of 1,004 community-dwelling adults across the lifespan. *J Psychiatr Res.* Aug 2022;152:160-166.

***Reason for exclusion:*** Did not meet the inclusion criteria (population).

Almeida OP, Patel H, Velasquez D, et al. Behavioral Activation in Nursing Homes to Treat Depression (BAN-Dep): Results From a Clustered, Randomized, Single-Blinded, Controlled Clinical Trial. *Am J Geriatr Psychiatry.* May 18 2022;18:18.

***Reason for exclusion:*** Did not meet the inclusion criteria (population).

Antoine V, Edy T, Souid M, Barthelemy F, Saint-Jean O. [Concerning: aging, the beginning of dialysis, the beginning of dependence: repercussions on the psychopathology of the very old dialysis patient]. *Nephrologie.* 2004;25(3):83-88.

***Reason for exclusion:*** Did not meet the inclusion criteria (not a research article).

Arosio B, Ostan R, Mari D, et al. Cognitive status in the oldest old and centenarians: a condition crucial for quality of life methodologically difficult to assess. *Mech Ageing Dev.* Jul 2017;165(Pt B):185-194.

***Reason for exclusion:*** Did not meet the inclusion criteria (concept).

Baltes PB, Mayer KU. The Berlin Aging Study: Aging from 70 to 100. *The Berlin Aging Study: Aging from 70 to 100*. New York, NY: Cambridge University Press; US; 1999: xii, 552.

***Reason for exclusion:*** Did not meet the inclusion criteria (concept).

Barak Y, Leitch S, Glue P. The Great Escape. Centenarians' exceptional health. *Aging Clin Exp Res.* Mar 2021;33(3):513-520.

***Reason for exclusion:*** Did not meet the inclusion criteria (concept).

Barnow S, Linden M. [Psychosocial Risk Factors of the Wish to be Dead in the Elderly]. *Fortschr Neurol Psychiatr.* Apr 2002;70(4):185-191.

***Reason for exclusion:*** Did not meet the inclusion criteria (population).

Barnow S, Linden M, Freyberger HJ. The relation between suicidal feelings and mental disorders in the elderly: results from the Berlin Aging Study (BASE). *Psychol Med.* May 2004;34(4):741-746.

***Reason for exclusion:*** Did not meet the inclusion criteria (population).

Bauman A, Merom D, Bull FC, Buchner DM, Fiatarone Singh MA. Updating the Evidence for Physical Activity: Summative Reviews of the Epidemiological Evidence, Prevalence, and Interventions to Promote "Active Aging". *Gerontologist.* Apr 2016;56 Suppl 2:S268-280.

***Reason for exclusion:*** Did not meet the inclusion criteria (population).

Baztán JJ, Pérez Martínez DA, Fernández Alonso M, Aguado Ortego R, Bellando Álvarez G, de la Fuente González AM. Factores pronósticos de recuperación funcional en pacientes muy ancianos con ictus. Estudio de seguimiento al año. *Revista de Neurología.* 2007;44(10):577-583.

***Reason for exclusion:*** Did not meet the inclusion criteria (population).

Bergdahl E, Allard P, Alex L, Lundman B, Gustafson Y. Gender differences in depression among the very old. *Int Psychogeriatr.* Dec 2007;19(6):1125-1140.

***Reason for exclusion:*** Did not meet the inclusion criteria (population).

Bergdahl E, Allard P, Gustafson Y. Depression among the very old with dementia. *Int Psychogeriatr.* Jun 2011;23(5):756-763.

***Reason for exclusion:*** Did not meet the inclusion criteria (population).

Bergdahl E, Gustavsson JM, Kallin K, et al. Depression among the oldest old: the Umea 85+ study. *Int Psychogeriatr.* Dec 2005;17(4):557-575.

***Reason for exclusion:*** Did not meet the inclusion criteria (concept).

Berlau DJ, Corrada MM, Peltz CB, Kawas CH. Disability in the oldest-old: incidence and risk factors in the 90+ study. *Am J Geriatr Psychiatry.* Feb 2012;20(2):159-168.

***Reason for exclusion:*** Did not meet the inclusion criteria (population).

Bishop AJ, Martin P, Randall GK, MacDonald M, Poon L. Exploring Life Satisfaction in Exceptional Old Age: The Mediating Role of Positive and Negative Affect. *Clin Gerontol.* 2012;35(2):105-125.

***Reason for exclusion:*** Did not meet the inclusion criteria (concept).

Blazer D, Burchett B, Service C, George LK. The association of age and depression among the elderly: an epidemiologic exploration. *J Gerontol.* Nov 1991;46(6):M210-215.

***Reason for exclusion:*** Did not meet the inclusion criteria (population).

Blazer DG. Psychiatry and the oldest old. *Am J Psychiatry.* Dec 2000;157(12):1915-1924.

***Reason for exclusion:*** Did not meet the inclusion criteria (population).

Boffelli S, Cassinadri A, Mercurio F, Rozzini R, Trabucchi M. Post hospital sub acute care: A new model of caring through geriatric methodology. *Giornale di Gerontologia.* 2014;62(1):21-28.

***Reason for exclusion:*** Did not meet the inclusion criteria (population).

Brandstetter K, Ahmadi M, O'Connor K, et al. Depression and Frailty in Community Dwelling Octogenarians, Nonagenarians and Centenarians K. Brandstetter1, M. Ahmadi1, K. O'Connor1, L. Evans1, J. Ceimo1 D. Coon2, and W. Nieri1 1BSHRI Center for Healthy Aging, Sun City, AZ; 2College of Nursing and Health Innovation, AZ State University, Phoenix, AZ. *Journal of the American Geriatrics Society.* Apr 2013;61:S163-S163.

***Reason for exclusion:*** Did not meet the inclusion criteria (concept).

Calderon KS. Making the Connection Between Depression and Activity Levels Among the Oldest-Old. *Activities, Adaptation & Aging.* 2001;25(2):59-73.

***Reason for exclusion:*** Did not meet the inclusion criteria (population).

Cheng, A., Leung, Y., Harrison, F., & Brodaty, H. (2019). The prevalence and predictors of anxiety and depression in near-centenarians and centenarians: a systematic review [Systematic Review]. International Psychogeriatrics, 31(11), 1539-1558. https://doi.org/10.1017/S1041610219000802

***Reason for exclusion:*** Did not meet the inclusion criteria (concept).

Cho J, Copeland LA, Stock EM, et al. Protective and Risk Factors for 5-Year Survival in the Oldest Veterans: Data from the Veterans Health Administration. *J Am Geriatr Soc.* Jun 2016;64(6):1250-1257.

***Reason for exclusion:*** Did not meet the inclusion criteria (concept).

Cho J, Martin P, Margrett J, MacDonald M, Poon LW, Johnson MA. Cohort comparisons in resources and functioning among centenarians: Findings from the Georgia Centenarian Study. International Journal of Behavioral Development. 2012;36(4):271-8.

***Reason for exclusion:*** Did not meet the inclusion criteria (outcome).

Christensen K, McGue M, Petersen I, Jeune B, Vaupel JW. Exceptional longevity does not result in excessive levels of disability. *Proc Natl Acad Sci U S A.* Sep 9 2008;105(36):13274-13279.

***Reason for exclusion:*** Did not meet the inclusion criteria (concept).

Cicconetti P, Tafaro L, Tedeschi G, Tombolillo MT, Marigliano V. [Cardiovascular risk factors and diseases in centenarians]. *Recenti Prog Med.* Dec 2001;92(12):731-734.

***Reason for exclusion:*** Did not meet the inclusion criteria (concept).

Da Rosa GD, Martin P, Margrett J. Functional Capacity and Personality as Predictors of Depressive Symptoms Among Centenarians. *Gerontologist.* Oct 2010;50:103-104.

***Reason for exclusion:*** Did not meet the inclusion criteria (abstract).

Deiana L, Ferrucci L, Pes GM, Carru C, Delitala G, Ganau A, et al. AKEntAnnos. The Sardinia Study of Extreme Longevity. *Aging* (Milano). 1999;11(3):142-9.

***Reason for exclusion:*** Did not meet the inclusion criteria (outcome).

Demosthenes B Panagiotakos DB, Chrysohoou C, Siassos G, et al. Poster session 3: The secrets of the very elderly: the Ikaria Island study. *European Journal of Cardiovascular Prevention & Rehabilitation.* 2011;18(1_suppl):S67-S91.

***Reason for exclusion:*** Did not meet the inclusion criteria (abstract).

Drageset J, Eide GE, Ranhoff AH. Cancer Diagnosis and Other Risk Factors for Hospitalization in Cognitive Intact Nursing Home Residents: A 5 Years Follow-Up Study. *Psycho-Oncology.* Oct 2014;23:398-399.

***Reason for exclusion:*** Did not meet the inclusion criteria (abstract).

Duarte N, Teixeira L, Ribeiro O, Paul C. Frailty phenotype criteria in centenarians: Findings from the Oporto Centenarian Study. *European Geriatric Medicine*. 2014;5(6):371-6.

***Reason for exclusion:*** Did not meet the inclusion criteria (concept).

Elinge E, Stenvall M, von Heideken Wagert P, Lofgren B, Gustafson Y, Nyberg L. Daily life among the oldest old with and without previous hip fractures. *Scand J Occup Ther.* Jun 2005;12(2):51-58.

***Reason for exclusion:*** Did not meet the inclusion criteria (population).

Etxeberria I, Etxebarria I, Urdaneta E. Profiles in emotional aging: does age matter? *Aging Ment Health.* Oct 2018;22(10):1304-1312.

***Reason for exclusion:*** Did not meet the inclusion criteria (concept).

Fauth EB, Gerstorf D, Ram N, Malmberg B. Comparing changes in late-life depressive symptoms across aging, disablement, and mortality processes. *Dev Psychol.* May 2014;50(5):1584-1593.

***Reason for exclusion:*** Did not meet the inclusion criteria (population).

Ferreira Agreli B, Aparecida Dias F, Dos Santos Ferreira PC, Candida Gomes N, Dos Santos Tavares DM. Functional disability and morbidities among the elderly people, according to socio-demographic conditions and indicative of depression. *Invest Educ Enferm.* Jan 2017;35(1):48-58.

***Reason for exclusion:*** Did not meet the inclusion criteria (population).

Forsell Y, Jorm AF, von Strauss E, Winblad B. Prevalence and correlates of depression in a population of nonagenarians. Br J Psychiatry. 1995;167(1):61-4.

***Reason for exclusion:*** Did not meet the inclusion criteria (outcome).

Gondo Y, Hirose N, Arai Y, et al. Contribution of an affect-associated gene to human longevity: prevalence of the long-allele genotype of the serotonin transporter-linked gene in Japanese centenarians. *Mech Ageing Dev.* Nov 2005;126(11):1178-1184.

***Reason for exclusion:*** Did not meet the inclusion criteria (concept).

Hajek A, Brettschneider C, Luhmann D, et al. [Does an Increase in Depressive Symptoms Lead to an Increase in Functional Impairment or vice versa? Results of the AgeCoDe and AgeQualiDe Study]. *Psychiatr Prax.* Apr 2020;47(3):148-153.

***Reason for exclusion:*** Did not meet the inclusion criteria (population).

Hajek A, Brettschneider C, Mallon T, et al. Depressive Symptoms and Frailty Among the Oldest Old: Evidence from a Multicenter Prospective Study. *J Am Med Dir Assoc.* Mar 2021;22(3):577-582 e572.

***Reason for exclusion:*** Did not meet the inclusion criteria (population).

Halaschek-Wiener J, Tindale LC, Collins JA, et al. The Super-Seniors Study: Phenotypic characterization of a healthy 85+ population. *PLoS ONE.* 2018;13(5):e0197578.

***Reason for exclusion:*** Did not meet the inclusion criteria (population).

Hall WJ. Centenarians: metaphor becomes reality. *Arch Intern Med.* Feb 11 2008;168(3):262-263.

***Reason for exclusion:*** Did not meet the inclusion criteria (not a research article).

Han K, Yang S, Jia W, et al. Health-Related Quality of Life and Its Correlation With Depression Among Chinese Centenarians. *Front Public Health.* 2020;8:580757.

***Reason for exclusion:*** Did not meet the inclusion criteria (concept).

Hartvigsen J, Christensen K. Pain in the back and neck are with us until the end: a nationwide interview-based survey of Danish 100-year-olds. *Spine (Phila Pa 1976).* Apr 15 2008;33(8):909-913.

***Reason for exclusion:*** Did not meet the inclusion criteria (concept).

Hazra NC, Dregan A, Jackson S, Gulliford MC. Differences in Health at Age 100 According to Sex: Population-Based Cohort Study of Centenarians Using Electronic Health Records. *J Am Geriatr Soc.* Jul 2015;63(7):1331-1337.

***Reason for exclusion:*** Did not meet the inclusion criteria (concept).

He Y, Zhao Y, Yang S. Cohort Study of Centenarians in Hainan, China (CHCCS). *Encyclopedia of Gerontology and Population Aging*. NA2019. p. 1-8.

***Reason for exclusion:*** Did not meet the inclusion criteria (outcome).

Heidrich SM. The relationship between physical health and psychological well-being in elderly women: a developmental perspective. *Res Nurs Health.* Apr 1993;16(2):123-130.

***Reason for exclusion:*** Did not meet the inclusion criteria (population).

Hensley B, Martin P, MacDonald M, et al. Family history and adaptation among centenarians and octogenarians. *Gerontology.* 2010;56(1):83-87.

***Reason for exclusion:*** Did not meet the inclusion criteria (concept).

Herr M, Arvieu JJ, Robine JM, Ankri J. Health, frailty and disability after ninety: Results of an observational study in France. *Arch Gerontol Geriatr.* Sep-Oct 2016;66:166-175.

***Reason for exclusion:*** Did not meet the inclusion criteria (population).

Hornsten C, Lovheim H, Gustafson Y. The association between stroke, depression, and 5-year mortality among very old people. *Stroke.* Sep 2013;44(9):2587-2589.

***Reason for exclusion:*** Did not meet the inclusion criteria (population).

Hornsten C, Molander L, Gustafson Y. The prevalence of stroke and the association between stroke and depression among a very old population. *Arch Gerontol Geriatr.* Nov-Dec 2012;55(3):555-559.

***Reason for exclusion:*** Did not meet the inclusion criteria (concept).

Hybels CF, Burchett B, Fillenbaum GG, Blazer D. Risk factors that predict extraordinary survival among older adults: The complex association between depressive symptoms and mortality. *American Journal of Geriatric Psychiatry.* Mar 2012;20(3):S121-S122.

***Reason for exclusion:*** Did not meet the inclusion criteria (abstract).

Izawa S, Hasegawa J, Enoki H, Iguch A, Kuzuya M. Depressive symptoms of informal caregivers are associated with those of community-dwelling dependent care recipients. *Int Psychogeriatr.* Dec 2010;22(8):1310-1317.

***Reason for exclusion:*** Did not meet the inclusion criteria (population).

Johnson MA, Brown MA, Poon LW, Martin P, Clayton GM. Nutritional patterns of centenarians. *Int J Aging Hum Dev*. 1992;34(1):57-76.

***Reason for exclusion:*** Did not meet the inclusion criteria (concept).

Johnson MCN. Examining resources and their influence on developmental health outcomes in older African Americans. *Dissertation Abstracts International Section A: Humanities and Social Sciences.* 2016;77(6-A(E)):No Pagination Specified.

***Reason for exclusion:*** Did not meet the inclusion criteria (concept).

Jones RN, Marcantonio ER, Rabinowitz T. Prevalence and correlates of recognized depression in U.S. nursing homes. *J Am Geriatr Soc.* Oct 2003;51(10):1404-1409.

***Reason for exclusion:*** Did not meet the inclusion criteria (concept).

Jopp DS, Boerner K, Rott C. Health and Disease at Age 100: Findings From the Second Heidelberg Centenarian Study. Deutsches Ärzteblatt international. 2016;113(12):203-10.

***Reason for exclusion:*** Did not meet the inclusion criteria (concept).

Jopp DS, Hicks S. Fordham Centenarian Study. *Encyclopedia of Geropsychology*. NA2016. p. 1-9.

***Reason for exclusion:*** Repetition (description of findings already included in this scoping review).

Jorge LB, Neves BB, Rocha JD, Ulrich V, Bos AJG. Impact of self-perceived health on long-lived subjects with urinary incontinence. *Scientia Medica.* 2019;29(1).

***Reason for exclusion:*** Did not meet the inclusion criteria (population).

Jung YH, Lee S, Kim WJ, Lee JH, Kim MJ, Han HJ. Effect of Integrated Cognitive Intervention Therapy in Patients with Mild to Moderate Alzheimer's Disease. *Dement Neurocogn Disord.* Sep 2020;19(3):86-95.

***Reason for exclusion:*** Did not meet the inclusion criteria (population).

Kato K, Zweig R, Schechter CB, Barzilai N, Atzmon G. Positive attitude toward life, emotional expression, self-rated health, and depressive symptoms among centenarians and near-centenarians. *Aging Ment Health.* Sep 2016;20(9):930-939.

***Reason for exclusion:*** Did not meet the inclusion criteria (concept).

Kheirbek RE, Fokar A, Wilson-Bell L, DeGrote S. Life at the extreme: Characteristics of veteran centenarians in long-term care. *Annals of Long-Term Care.* 2018;26(5):E25-E32.

***Reason for exclusion:*** Did not meet the inclusion criteria (concept).

Kulminski AM, Arbeev KG, Christensen K, et al. Do gender, disability, and morbidity affect aging rate in the LLFS? Application of indices of cumulative deficits. *Mech Ageing Dev.* Apr 2011;132(4):195-201.

***Reason for exclusion:*** Did not meet the inclusion criteria (concept).

Lau BH, Kwan JS, Cheung KS, Martin P. Depression Moderates the Frailty-Subjective Health Link among Chinese Near Centenarians and Centenarians. *Am J Geriatr Psychiatry.* Sep 2016;24(9):753-761.

***Reason for exclusion:*** Did not meet the inclusion criteria (concept).

Lee G, Arieli R, Ryou YJ, Martin P. The bidirectional relationship between depressive symptoms and functional limitations among centenarian survivors in their 80s: Testing bivariate latent change score models. *Aging Ment Health*. 2023:1-9.

***Reason for exclusion:*** Did not meet the inclusion criteria (population).

Legrand R, Manckoundia P, Nuemi G, Poulain M. Assessment of the Health Status of the Oldest Olds Living on the Greek Island of Ikaria: A Population Based-Study in a Blue Zone. *Curr Gerontol Geriatr Res.* 2019;2019:8194310.

***Reason for exclusion:*** Did not meet the inclusion criteria (population).

Leitch S, Glue P, Gray AR, Greco P, Barak Y. Comparison of Psychosocial Variables Associated With Loneliness in Centenarian vs Elderly Populations in New Zealand. *JAMA Netw Open.* Oct 5 2018;1(6):e183880.

***Reason for exclusion:*** Did not meet the inclusion criteria (concept).

Linden M, Horgas AL, Gilberg R, Steinhagen-Thiessen E. Predicting health care utilization in the very old. The role of physical health, mental health, attitudinal and social factors. *J Aging Health.* Feb 1997;9(1):3-27.

***Reason for exclusion:*** Did not meet the inclusion criteria (population).

Lisko I, Tormakangas T, Jylha M. Structure of self-rated health among the oldest old: Analyses in the total population and those living with dementia. *SSM Popul Health.* Aug 2020;11:100567.

***Reason for exclusion:*** Did not meet the inclusion criteria (population).

MacDonald M, Martin P, Margrett J, Poon LW. Correspondence of perceptions about centenarians' mental health. *Aging Ment Health*. 2009;13(6):827-37.

***Reason for exclusion:*** Did not meet the inclusion criteria (concept).

Margrett JA, Daugherty K, Martin P, et al. Affect and loneliness among centenarians and the oldest old: the role of individual and social resources. *Aging Ment Health.* Apr 2011;15(3):385-396.

***Reason for exclusion:*** Did not meet the inclusion criteria (concept).

Motta M, Bennati E, Ferlito L, Malaguarnera M, Motta L, Italian Multicenter Study on C. Successful aging in centenarians: myths and reality. *Arch Gerontol Geriatr.* May-Jun 2005;40(3):241-251.

***Reason for exclusion:*** Did not meet the inclusion criteria (concept).

Niimura H, Eguchi Y, Kida H, Nishimura W, Takayama M, Mimura M. Cohort study of the oldest-old of Japan: The Arakawa 95+ study. *European Geriatric Medicine.* 2017;8:S197-S198.

***Reason for exclusion:*** Did not meet the inclusion criteria (abstract).

Niklasson J, Hornsten C, Conradsson M, et al. High morale is associated with increased survival in the very old. *Age Ageing.* Jul 2015;44(4):630-636.

***Reason for exclusion:*** Did not meet the inclusion criteria (concept).

Nybo H, Gaist D, Jeune B, et al. The Danish 1905 cohort: a genetic-epidemiological nationwide survey. *J Aging Health.* Feb 2001;13(1):32-46.

***Reason for exclusion:*** Did not meet the inclusion criteria (population).

Nygren B, Alex L, Jonsen E, Gustafson Y, Norberg A, Lundman B. Resilience, sense of coherence, purpose in life and self-transcendence in relation to perceived physical and mental health among the oldest old. *Aging Ment Health.* Jul 2005;9(4):354-362.

***Reason for exclusion:*** Did not meet the inclusion criteria (concept).

Odden MC, Koh WJH, Arnold AM, Rawlings AM, Psaty BM, Newman AB. Trajectories of Nonagenarian Health: Sex, Age, and Period Effects. *Am J Epidemiol.* Feb 1 2019;188(2):382-388.

***Reason for exclusion:*** Did not meet the inclusion criteria (population).

Ogliari G, Mari D, Ronchetti F, et al. Gender-differences in centenarians: Health status and life-style. *European Geriatric Medicine.* 2013;4:S122.

***Reason for exclusion:*** Did not meet the inclusion criteria (abstract).

Pioggiosi P, Forti P, Ravaglia G, Berardi D, Ferrari G, De Ronchi D. Different classification systems yield different dementia occurrence among nonagenarians and centenarians. *Dement Geriatr Cogn Disord.* Dec 2004;17(1-2):35-41.

***Reason for exclusion:*** Did not meet the inclusion criteria (concept).

Pioggiosi PP, Berardi D, Ferrari B, Quartesan R, De Ronchi D. Occurrence of cognitive impairment after age 90: MCI and other broadly used concepts. *Brain Res Bull.* Jan 15 2006;68(4):227-232.

***Reason for exclusion:*** Did not meet the inclusion criteria (concept).

Poon LW. What can we learn from centenarians? [References]. *Aging, biotechnology and the future*. Baltimore, MD: Johns Hopkins University Press; US; 2008: 100-110.

***Reason for exclusion:*** Did not meet the inclusion criteria (concept).

Powell AL. On the Question of the Mental State of Centenarians. *Dementia.* May-Jun 1992;3(3):150-156.

***Reason for exclusion:*** Did not meet the inclusion criteria (concept).

Rasmussen SH, Andersen-Ranberg K, Thinggaard M, et al. Cohort Profile: The 1895, 1905, 1910 and 1915 Danish Birth Cohort Studies - secular trends in the health and functioning of the very old. *Int J Epidemiol.* Dec 1 2017;46(6):1746-1746j.

***Reason for exclusion:*** Did not meet the inclusion criteria (concept).

Receputo G, Rapisarda R, Motta L. [Centenarians: health status and life conditions]. *Ann Ital Med Int.* Jan-Mar 1995;10(1):41-45.

***Reason for exclusion:*** Did not meet the inclusion criteria (no methods section).

Ribeiro O, Araujo L, Teixeira L, Duarte N, Brandao D, Martin I, et al. Health Status, Living Arrangements, and Service Use at 100: Findings From the Oporto Centenarian Study*. J Aging Soc Policy*. 2016;28(3):148-64.

***Reason for exclusion:*** Did not meet the inclusion criteria (concept).

Rigo, II, Bos AJG. [Family dysfunction in nonagenarians and centenarians: the importance of health conditions and social support]. *Cien Saude Colet.* 2021;26(6):2355-2364.

***Reason for exclusion:*** Did not meet the inclusion criteria (population).

Rozzini R, Sleiman I, Maggi S, Noale M, Trabucchi M. Gender differences and health status in old and very old patients. *J Am Med Dir Assoc.* Oct 2009;10(8):554-558.

***Reason for exclusion:*** Did not meet the inclusion criteria (population).

Ruiz JR, Gil-Bea F, Bustamante-Ara N, et al. Resistance training does not have an effect on cognition or related serum biomarkers in nonagenarians: a randomized controlled trial. *Int J Sports Med.* Jan 2015;36(1):54-60.

***Reason for exclusion:*** Did not meet the inclusion criteria (population).

Sachdev PS, Levitan C, Crawford J, et al. The Sydney Centenarian Study: methodology and profile of centenarians and near-centenarians. *Int Psychogeriatr.* Jun 2013;25(6):993-1005.

***Reason for exclusion:*** Did not meet the inclusion criteria (concept).

Samuelsson SM, Alfredson BB, Hagberg B, Samuelsson G, Nordbeck B, Brun A, et al. The Swedish Centenarian Study: a multidisciplinary study of five consecutive cohorts at the age of 100. *Int J Aging Hum Dev*. 1997;45(3):223-53.

***Reason for exclusion:*** Did not meet the inclusion criteria (concept).

Scelzo A, Di Somma S, Antonini P, et al. Mixed-methods quantitative-qualitative study of 29 nonagenarians and centenarians in rural Southern Italy: focus on positive psychological traits. *Int Psychogeriatr.* Jan 2018;30(1):31-38.

***Reason for exclusion:*** Did not meet the inclusion criteria (population).

Scheetz LT, Martin P, Poon LW. Do centenarians have higher levels of depression? Findings from the Georgia Centenarian Study. *J Am Geriatr Soc.* Feb 2012;60(2):238-242.

***Reason for exclusion:*** Did not meet the inclusion criteria (concept).

Selim AJ, Fincke G, Berlowitz DR, Miller DR, Qian SX, Lee A, et al. Comprehensive health status assessment of centenarians: results from the 1999 large health survey of veteran enrollees. *J Gerontol A Biol Sci Med Sci*. 2005;60(4):515-9.

***Reason for exclusion:*** Did not meet the inclusion criteria (concept).

Spini D, Ghisletta P. Dynamics between depression and functional health in the swiss interdisciplinary longitudinal study on the oldest old. *Gerontologist.* Oct 2003;43:458-458.

***Reason for exclusion:*** Did not meet the inclusion criteria (abstract).

Strinnholm S, Gustafson Y, Niklasson J. Depressive Disorders and Religious Engagement in Very Old People. *Gerontol Geriatr Med.* Jan-Dec 2019;5:2333721419846576.

***Reason for exclusion:*** Did not meet the inclusion criteria (concept).

Szczerbinska K, Hirdes JP, Zyczkowska J. Good news and bad news: depressive symptoms decline and undertreatment increases with age in home care and institutional settings. *Am J Geriatr Psychiatry.* Dec 2012;20(12):1045-1056.

***Reason for exclusion:*** Did not meet the inclusion criteria (concept).

Tafaro L, Tombolillo MT, Brukner N, et al. Stress in centenarians. *Arch Gerontol Geriatr.* May-Jun 2009;48(3):353-355.

***Reason for exclusion:*** Did not meet the inclusion criteria (not a research article).

Tan CL, Wang H, Zhang GF. Abstracts from the Seventh Chinese Congress on Gerontology and Health Industry. *J Am Geriatr Soc.* Sep 2018;66 Suppl 3:S443-S514.

***Reason for exclusion:*** Did not meet the inclusion criteria (abstract).

Tanprasertsuk J, Johnson EJ, Johnson MA, et al. Clinico-Neuropathological Findings in the Oldest Old from the Georgia Centenarian Study. *J Alzheimers Dis.* 2019;70(1):35-49.

***Reason for exclusion:*** Did not meet the inclusion criteria (concept).

Tanprasertsuk JBB. Fat-soluble nutrients in serum and brain of centenarians and their relationship to neuropathology and cognition. *Dissertation Abstracts International: Section B: The Sciences and Engineering.* 2019;80(8-B(E)):No Pagination Specified.

***Reason for exclusion:*** Did not meet the inclusion criteria (concept).

Teixeira C, Nunes F, Ribeiro F, Arbinaga F, Vasconcelos-Raposo J. Physical activity, self-esteem and depression in older adults. *Cuadernos de Psicologia del Deporte.* 2016;16(3):55-66.

***Reason for exclusion:*** Did not meet the inclusion criteria (population).

Tettamanti M, Recchia A, Garrì M, et al. P2‐166: Walking in late middle age and prevalence of dementia in centenarians in the monzino 80‐plus study. *Alzheimer's & Dementia.* 2011;7(4S_Part_10):S365.

***Reason for exclusion:*** Did not meet the inclusion criteria (abstract).

von Heideken Wagert P, Gustafson Y, Kallin K, Jensen J, Lundin-Olsson L. Falls in very old people: the population-based Umea 85+ study in Sweden. *Arch Gerontol Geriatr.* Nov-Dec 2009;49(3):390-396.

***Reason for exclusion:*** Did not meet the inclusion criteria (population).

von Heideken Wagert P, Ronnmark B, Rosendahl E, et al. Morale in the oldest old: the Umea 85+ study. *Age Ageing.* May 2005;34(3):249-255.

***Reason for exclusion:*** Did not meet the inclusion criteria (concept).

von Strauss E, Fratiglioni L, Viitanen M, Forsell Y, Winblad B. Morbidity and comorbidity in relation to functional status: a community-based study of the oldest old (90+ years). *J Am Geriatr Soc.* Nov 2000;48(11):1462-1469.

***Reason for exclusion:*** Did not meet the inclusion criteria (concept).

Wang H, Wang Q, Wu XL, Liu GD, Li CF. Assessment of Health and Spectrum of Diseases in Centenarians in Zhongxiang, Hubei Province. *Journal of the American Geriatrics Society.* Sep 2016;64:S359-S360.

***Reason for exclusion:*** Did not meet the inclusion criteria (paper session).

Weidung B, Toots A, Nordstrom P, Carlberg B, Gustafson Y. Systolic blood pressure decline in very old individuals is explained by deteriorating health: Longitudinal changes from Umea85+/GERDA. *Medicine (Baltimore).* Dec 2017;96(51):e9161.

***Reason for exclusion:*** Did not meet the inclusion criteria (population).

Whittle C, Corrada MM, Dick M, et al. Neuropsychological data in nondemented oldest old: the 90+ Study. *J Clin Exp Neuropsychol.* Apr 2007;29(3):290-299.

***Reason for exclusion:*** Did not meet the inclusion criteria (concept).

Wu H, Flaherty J, Dong B, Liu G, Deng J, Zhang Y, et al. Impact of geriatric conditions versus medical diagnoses on ADL disability among nonagenarians and centenarians. *J Aging Health*. 2012;24(8):1298-319.

***Reason for exclusion:*** Did not meet the inclusion criteria (concept).

Wu YG, Zhang GB, Zhang CL, Li ZB. Mental health investigation of people over 100 years old in Bama County. *Journal of Clinical Rehabilitative Tissue Engineering Research.* 2007;11(52):10572-10575.

***Reason for exclusion:*** Did not meet the inclusion criteria (written in Chinese; untranslatable format).

Xu X, Zhao Y, Xia SY, Cui PP, Tang WM, Hu XL, et al. Quality of Life and Its Influencing Factors Among Centenarians in Nanjing, China: A Cross-Sectional Study. *Social Indicators Research*. 2022;160(2-3):735-55.

***Reason for exclusion:*** Did not meet the inclusion criteria (outcome).

Yang S, Wang S, Liu W, et al. Malnutrition Is an Independent Risk Factor for Low Health-Related Quality of Life Among Centenarians. *Front Med (Lausanne).* 2021;8:729928.

***Reason for exclusion:*** Did not meet the inclusion criteria (concept).

Yao Y, Liu M, Yang SS, et al. [Study on self-rated health and related factors in centenarians in Hainan province]. *Zhonghua Liu Xing Bing Xue Za Zhi.* Mar 10 2018;39(3):264-267.

***Reason for exclusion:*** Did not meet the inclusion criteria (written in Chinese; untranslatable format).

Yu T. 2018 Annual Scientific Meeting: Weight loss in a nonagenarian female. *J Am Geriatr Soc.* Apr 2018;66 Suppl 2:S1-S369.

***Reason for exclusion:*** Did not meet the inclusion criteria (abstract).

Zhang GF, Wang H, Ye GM, Tan CL, Liu GD, Li CF. Abstracts from the Seventh Chinese Congress on Gerontology and Health Industry: Quality of life of longevously people in Zhongxiang City Hubei province.. *J Am Geriatr Soc.* Sep 2018;66 Suppl 3:S443-S514.

***Reason for exclusion:*** Did not meet the inclusion criteria (abstract).

Zhang Y, Xiong Y, Yu Q, Shen S, Chen L, Lei X. The activity of daily living (ADL) subgroups and health impairment among Chinese elderly: a latent profile analysis. *BMC geriatr.* Jan 7 2021;21(1):30.

***Reason for exclusion:*** Did not meet the inclusion criteria (population).

Zou C, Zhou Y, Dong B, Hao Q, Chen S, Zhou J. Predictors of 49-month mortality in Chinese nonagenarians and centenarians in PLAD study. *Aging Clin Exp Res.* Dec 2015;27(6):821-827.

***Reason for exclusion:*** Did not meet the inclusion criteria (population).
