# Supplementary material for: Depressive symptoms and functional dependence in near-centenarians and centenarians: a scoping review
Source: BMC Geriatr. 2026 Feb 6;26:321. doi: 10.1186/s12877-026-07026-4 (PMC12977654; doi:10.1186/s12877-026-07026-4)
Supplement: Supplementary file 3 — Additional file 3: Data extraction instrument. [file 12877_2026_7026_MOESM3_ESM.docx]

**Additional file 3.** Data extraction instrument

| **ID** | Author(s): |  |
| --- | --- | --- |
|  | Year of publication: |  |
|  | Country of origin: |  |
| **Methodological aspects (PCC)** | Methodological approach (quantitative, qualitative, mixed-methods): |  |
|  | Study design: |  |
|  | Study aim(s) / research questions(s) / hypothesis: |  |
|  | Population: | □ Near-centenarians (95–99 years old)  □ Centenarians (≥ 100 years old)  □ Near-centenarians and centenarians (≥ 95 years old) |
|  | Sample size: | *Fill in the relevant options:*   - General sample: n =______________________________________ - Subsample ≥ 95 years old: n =______________________________ - Subsample ≥ 95 and ≤ 99 years old: n =______________________ - Subsample ≥ 100 years old: n =_____________________________ |
|  | Mean or median age / age range: | *Fill in the relevant options:*   - General sample: X̅ or Md age =______________________________ - Subsample ≥ 95 years old: X̅ or Md age =______________________ - Subsample ≥ 95 and ≤ 99 years old: X̅ or Md age =______________ - Subsample ≥ 100 years old: X̅ or Md age =_____________________ - Age range / other: ______________________________________________ |
|  | Context: | □ Home: ____%  □ Nursing home or long-term care facility: ____%  □ Hospital (acute somatic setting): ____%  □ Hospital (psychiatric setting): ____%  □ Transitional care unit: ____%  □ Other: ____% |
|  | Concepts (relating to the phenomena of interest): |  |
|  | Instruments / strategies used to screen for depressive symptoms: |  |
|  | Is there a medical diagnosis of depression? | □ Yes  □ No |
|  | Instruments used to assess functional dependence: |  |
| **Main results** | Key findings relating to depressive symptoms: | □ Prevalence rate: _______________________________________  □ X̅ or MED score = _______________________________________  □ Other: _______________________________________________ |
|  | Key findings relating to functional dependence: | □ Prevalence rate: _______________________________________  □ X̅ or MED score = _______________________________________  □ Other: _______________________________________________ |
|  | Key findings relating to associations between depressive symptoms and functional dependence | □ Associations / relationships: __________________________________________________________________________________________________________________________________________________________________________________________________________________ |

**Additional comments:**
