# Supplementary material for: Depressive symptoms and functional dependence in near-centenarians and centenarians: a scoping review
Source: BMC Geriatr. 2026 Feb 6;26:321. doi: 10.1186/s12877-026-07026-4 (PMC12977654; doi:10.1186/s12877-026-07026-4)
Supplement: Supplementary file 4 — Additional file 4: Average number of studies by 5-year publication periods. [file 12877_2026_7026_MOESM4_ESM.docx]

**Additional file 4.** Average number of studies by 5-year publication periods (n = 53)


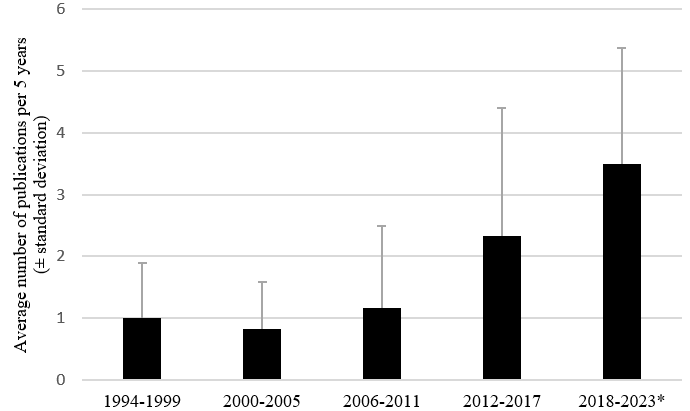


***** For the year 2023, only studies published up to August 28th were considered.
