# Supplementary material for: Depressive symptoms and functional dependence in near-centenarians and centenarians: a scoping review
Source: BMC Geriatr. 2026 Feb 6;26:321. doi: 10.1186/s12877-026-07026-4 (PMC12977654; doi:10.1186/s12877-026-07026-4)
Supplement: Supplementary file 5 — Additional file 5: Number of studies per country. [file 12877_2026_7026_MOESM5_ESM.docx]

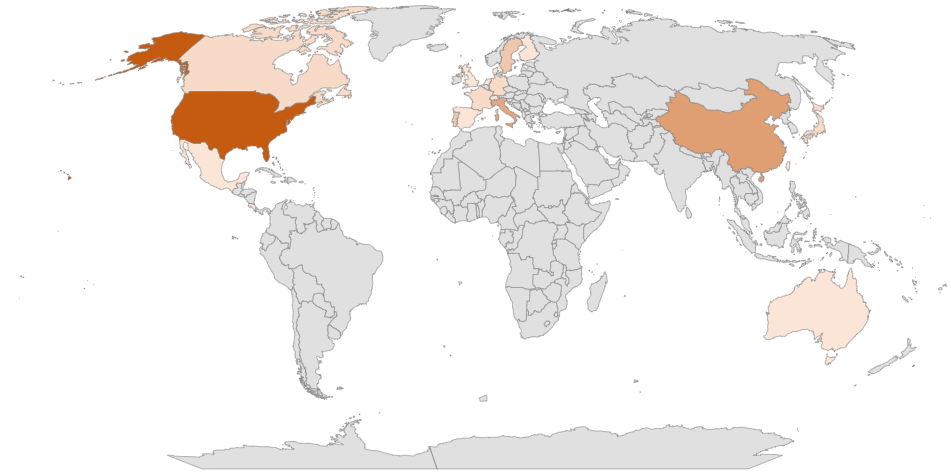

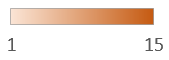
**Additional file 5.** Number of studies per country* (n = 53)

***** Three of the retained studies were part of international collaborations; each country involved is represented in the graph (e.g., for a collaboration between Portugal and Germany, both countries are represented).
