# Supplementary material for: Depressive symptoms and functional dependence in near-centenarians and centenarians: a scoping review
Source: BMC Geriatr. 2026 Feb 6;26:321. doi: 10.1186/s12877-026-07026-4 (PMC12977654; doi:10.1186/s12877-026-07026-4)
Supplement: Supplementary file 6 — Additional file 6: Visual representation of the prevalence of depressive symptoms. [file 12877_2026_7026_MOESM6_ESM.docx]

**Additional file 6.** Visual representation of the prevalence of depressive symptoms


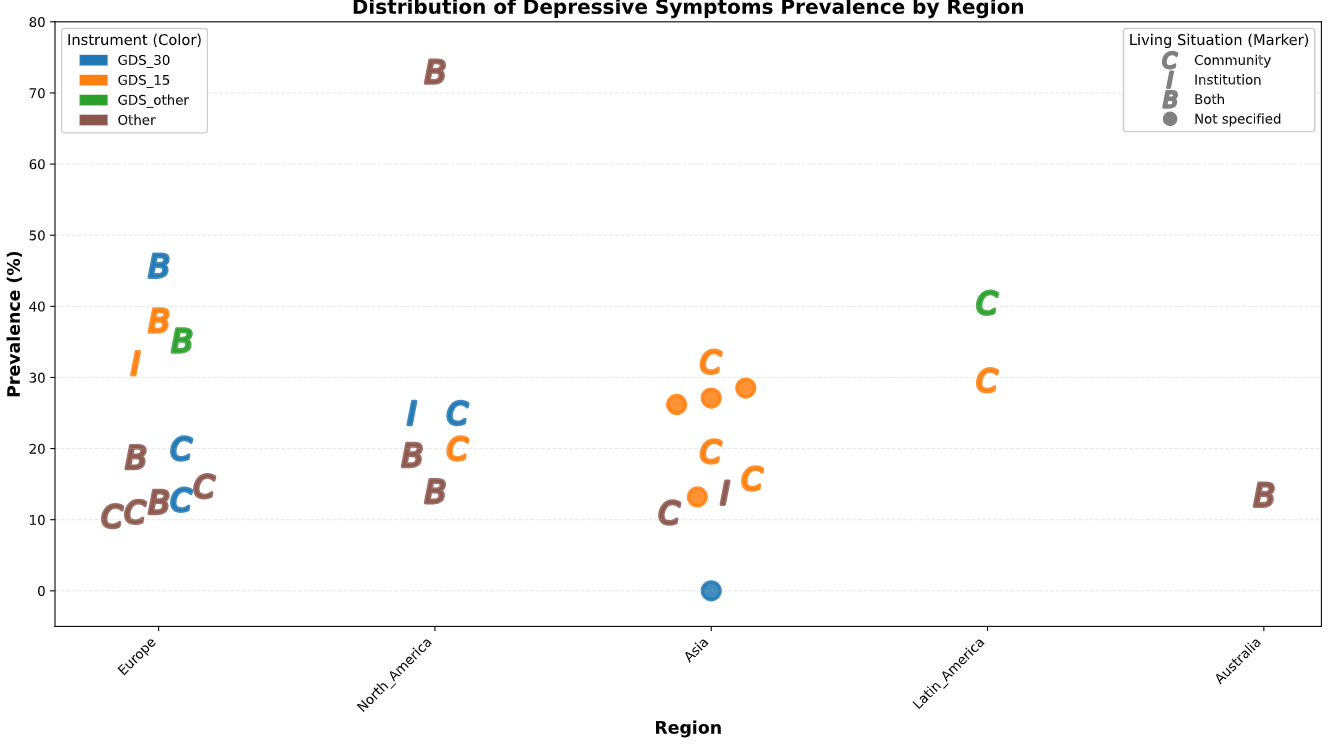


Note 1: For studies including participants from both settings, we applied a ≥70% cutoff to classify the sample as predominantly Community (C) or Institution (I). Samples not meeting this threshold were categorized as Both (B). For detailed information on living situation (specific proportions), please refer to Table 1.

Note 2: This figure presents depressive symptom prevalence rates from individual studies. Some of these rates were grouped for the analyses reported in the main text, which may explain any discrepancies.
