# Supplementary material for: Depressive symptoms and functional dependence in near-centenarians and centenarians: a scoping review
Source: BMC Geriatr. 2026 Feb 6;26:321. doi: 10.1186/s12877-026-07026-4 (PMC12977654; doi:10.1186/s12877-026-07026-4)
Supplement: Supplementary file 7 — Additional file 7: Identified instruments to screen for depressive symptoms. [file 12877_2026_7026_MOESM7_ESM.docx]

**Additional file 7.** Identified instruments to screen for depressive symptoms


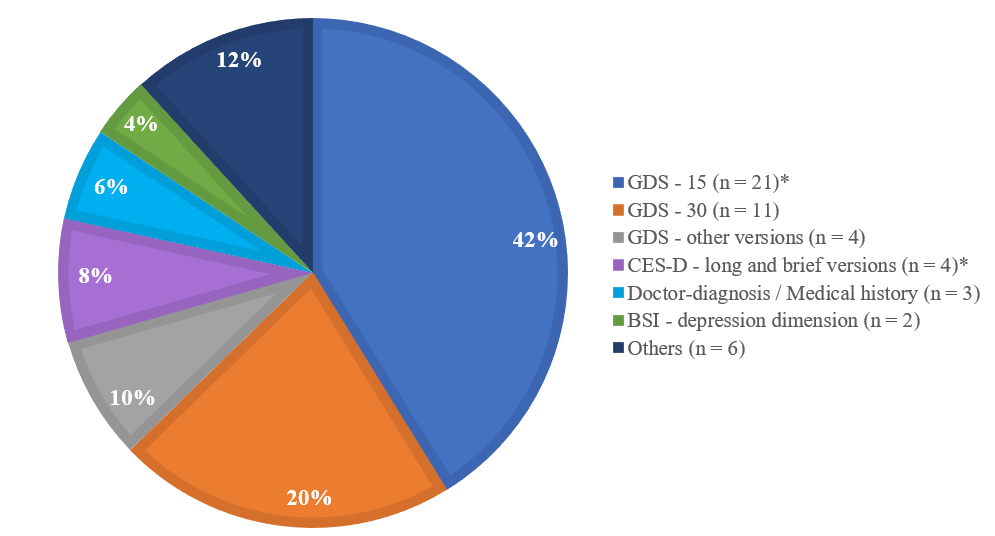
* The total is 102% due to the inclusion of the study by Niimura et al. (2020), which employed both the GDS-15 and the CES-D for screening depressive symptoms, and is therefore counted in both categories. Consequently, the chart represents 51 instances derived from 50 studies.
