# Supplementary material for: Depressive symptoms and functional dependence in near-centenarians and centenarians: a scoping review
Source: BMC Geriatr. 2026 Feb 6;26:321. doi: 10.1186/s12877-026-07026-4 (PMC12977654; doi:10.1186/s12877-026-07026-4)
Supplement: Supplementary file 9 — Additional file 9: Identified instruments to assess functional dependence. [file 12877_2026_7026_MOESM9_ESM.docx]

**
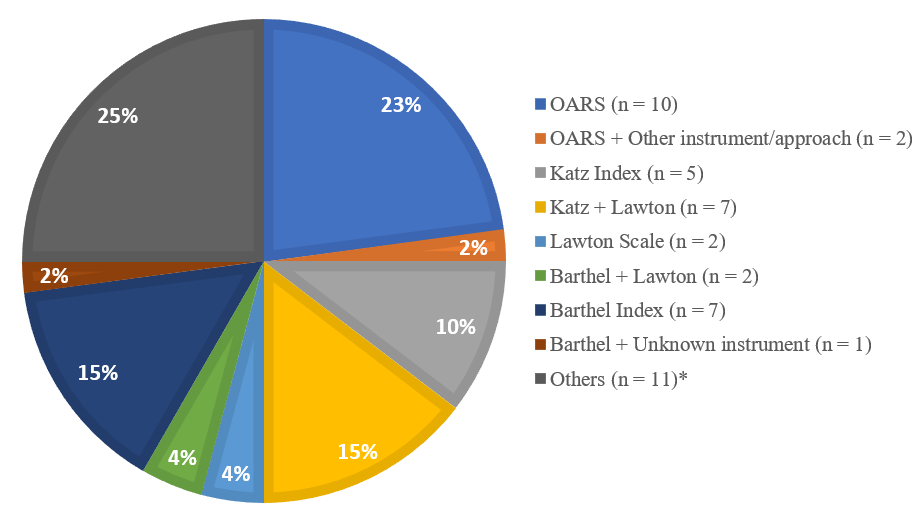
Additional file 9.** Identified instruments to assess functional dependence

*Other mapped instruments: The 13-question ADLs module; Cumulative scale (5 ADLs + 4 IADLs); DAFS-IADLs; DAFS-R; ADL-H; ADL-staircase; among others.

Note: Pie chart percentages represent the utilization of instruments either independently or in combination in some cases (e.g., the Katz Index was mapped in 12 (25.0%) different studies: it was used as a single instrument in five (10%), while in seven (15%) it was used in combination with the Lawton Scale).
