# Supplementary material for: Depressive symptoms and functional dependence in near-centenarians and centenarians: a scoping review
Source: BMC Geriatr. 2026 Feb 6;26:321. doi: 10.1186/s12877-026-07026-4 (PMC12977654; doi:10.1186/s12877-026-07026-4)
Supplement: Supplementary file 10 — Additional file 10: Summary of the main research gaps and future directions. [file 12877_2026_7026_MOESM10_ESM.docx]

**Additional file 10.** Summary of the main research gaps and future directions

| **Analysed domains** | **Identified gaps** | **Future directions** |
| --- | --- | --- |
| Study design | - Lack of longitudinal data on mental and functional health trajectories in very old age. - Minimal integration of qualitative techniques (only 3 studies). | - Longitudinal approaches could help capture both mental and functional health changes over time. - Qualitative approaches may offer important insights into age-specific nuances. - Mixed-methods designs might support a more comprehensive understanding. |
| Geographic diversity | - Studies mainly from North America, Europe, and Asia. - No studies from Africa or South America, and only one study from Australia. | - Expanding research to underrepresented regions, where feasible, may help fill current gaps. |
| Prevalence of depressive symptoms | - Wide variability in prevalence rates across studies. - Differences possibly partly explained by heterogeneity of instruments used. - Variability also in screening approaches, limiting comparability. | - Greater consistency in screening tools could enhance comparability across studies and regions. - Greater methodological consistency may help reduce variability. |
| Instruments used to assess depressive symptoms | - High diversity of instruments (10 tools identified). - Predominant use of GDS, but lack of hetero-assessment instruments (e.g., CSDD) despite relevance for those with major neurocognitive impairment. - No study used DSM or ICD criteria for diagnostic confirmation. - No screening tool validated specifically for near-centenarians and centenarians. | - Hetero-assessment tools are useful for individuals with major neurocognitive impairment and should be implemented. - Developing and testing a tool specifically for this age group could be beneficial. - The use of structured diagnostic criteria (e.g., DSM/ICD) should also be considered where feasible. |
| Prevalence of functional dependence | - Considerable variability in prevalence across studies for both ADLs and IADLs. - Variability possibly linked to differences in scoring strategies, even when using the same instrument. - Some studies assess only ADLs or only IADLs, limiting comparability. | - Greater consistency in screening tools could improve comparability across studies and regions. - Standardised scoring strategies may help reduce variation across studies. - Assessing both ADLs and IADLs consistently may strengthen cross-study alignment. |
| Instruments used to assess functional dependence | - Large number of instruments used (16 tools), creating high heterogeneity. - Instruments differ in focus (ADLs only, IADLs only, or both). - Variability in terminology (e.g., "functional dependence", "disability", graded categories). | - More harmonised measurement approaches could support consistency and comparability across studies. - Pairing tools to assess both ADLs and IADLs may offer a more complete picture when a single instrument does not cover both domains. - Clearer definitions and terminology could strengthen conceptual alignment. |
| Associations between depressive symptoms and functional dependence | - Only 16 out of 53 studies examined this association. - Mixed findings: some studies reported no significant correlation, others found significant associations. - Only a small number of studies analysed predictive relationships (n = 6). - Among predictive studies, results were inconsistent (some showing directional effects; others reporting no significant relationships). | - Further studies on the association between depressive symptoms and functional dependence in near-centenarians and centenarians are necessary. - Bidirectional relationships between depressive symptoms and functional dependence warrant further exploration. - Clarifying predictive pathways also require additional investigation. |

ADLs = Basic Activities of Daily Living (eating; dressing and undressing; grooming (taking care of own appearance); transferring (getting in and out of bed); walking; bathing or showering; using the toilet, including continence); IADLs = Instrumental Activities of Daily Living (using the telephone; managing transportation (getting to places out of walking distance); shopping for essentials; preparing meals; managing housekeeping; managing medications; handling finances); CSDD = Cornell Scale for Depression in Dementia; DSM = Diagnostic and Statistical Manual of Mental Disorders; GDS = Geriatric Depression Scale; ICD = International Classification of Diseases.
